# Genome-Wide Search for Gene-Gene Interactions in Colorectal Cancer

**DOI:** 10.1371/journal.pone.0052535

**Published:** 2012-12-26

**Authors:** Shuo Jiao, Li Hsu, Sonja Berndt, Stéphane Bézieau, Hermann Brenner, Daniel Buchanan, Bette J. Caan, Peter T. Campbell, Christopher S. Carlson, Graham Casey, Andrew T. Chan, Jenny Chang-Claude, Stephen Chanock, David V. Conti, Keith R. Curtis, David Duggan, Steven Gallinger, Stephen B. Gruber, Tabitha A. Harrison, Richard B. Hayes, Brian E. Henderson, Michael Hoffmeister, John L. Hopper, Thomas J. Hudson, Carolyn M. Hutter, Rebecca D. Jackson, Mark A. Jenkins, Elizabeth D. Kantor, Laurence N. Kolonel, Sébastien Küry, Loic Le Marchand, Mathieu Lemire, Polly A. Newcomb, John D. Potter, Conghui Qu, Stephanie A. Rosse, Robert E. Schoen, Fred R. Schumacher, Daniela Seminara, Martha L. Slattery, Cornelia M. Ulrich, Brent W. Zanke, Ulrike Peters

**Affiliations:** 1 Public Health Sciences Division, Fred Hutchinson Cancer Research Center, Seattle, Washington, United States of America; 2 Division of Cancer Epidemiology and Genetics, National Cancer Institute, Bethesda, Maryland, United States of America; 3 Service de Génétique Médicale, CHU Nantes, Nantes, France; 4 Division of Clinical Epidemiology and Aging Research, German Cancer Research Center, Heidelberg, Germany; 5 Cancer and Population Studies Group, Queensland Institute of Medical Research, Queensland, Australia; 6 Division of Research, Kaiser Permanente Medical Care Program, Oakland, California, United States of America; 7 Epidemiology Research Program, American Cancer Society, Atlanta, Georgia, United States of America; 8 School of Public Health, University of Washington, Seattle, Washington, United States of America; 9 Keck School of Medicine, University of Southern California, Los Angeles, California, United States of America; 10 Division of Gastroenterology, Massachusetts General Hospital and Harvard Medical School, Boston, Massachusetts, United States of America; 11 Channing Division of Network Medicine, Brigham and Women’s Hospital and Harvard Medical School, Boston, Massachusetts, United States of America; 12 Division of Cancer Epidemiology, German Cancer Research Center, Heidelberg, Germany; 13 Translational Genomics Research Institute, Phoenix, Arizona, United States of America; 14 Department of Surgery, University Health Network, Toronto General Hospital, Toronto, Canada; 15 Division of Epidemiology, New York University School of Medicine, New York, New York, United States of America; 16 Melbourne School of Population Health, University of Melbourne, Melbourne, Australia; 17 Ontario Institute for Cancer Research, Toronto, Canada; 18 Departments of Medical Biophysics and Molecular Genetics, University of Toronto, Toronto, Canada; 19 Division of Endocrinology, Diabetes, and Metabolism, Ohio State University, Columbus, Ohio, United States of America; 20 Epidemiology Program, University of Hawaii Cancer Center, Honolulu, Hawaii, United States of America; 21 Centre for Public Health Research, Massey University, Wellington, New Zealand; 22 Department of Medicine and Epidemiology, University of Pittsburgh Medical Center, Pittsburgh, Pennsylvania, United States of America; 23 Division of Cancer Control and Population Sciences, National Cancer Institute, Bethesda, Maryland, United States of America; 24 Department of Internal Medicine, University of Utah Health Sciences Center, Salt Lake City, Utah, United States of America; 25 Division of Preventive Oncology, National Center for Tumor Diseases and German Cancer Research Center, Heidelberg, Germany; 26 Clinical Epidemiology Program, Ottawa Hospital Research Institute, Ottawa, Canada; University of California, Irvine, United States of America

## Abstract

Genome-wide association studies (GWAS) have successfully identified a number of single-nucleotide polymorphisms (SNPs) associated with colorectal cancer (CRC) risk. However, these susceptibility loci known today explain only a small fraction of the genetic risk. Gene-gene interaction (GxG) is considered to be one source of the missing heritability. To address this, we performed a genome-wide search for pair-wise GxG associated with CRC risk using 8,380 cases and 10,558 controls in the discovery phase and 2,527 cases and 2,658 controls in the replication phase. We developed a simple, but powerful method for testing interaction, which we term the Average Risk Due to Interaction (ARDI). With this method, we conducted a genome-wide search to identify SNPs showing evidence for GxG with previously identified CRC susceptibility loci from 14 independent regions. We also conducted a genome-wide search for GxG using the marginal association screening and examining interaction among SNPs that pass the screening threshold (p<10^−4^). For the known locus rs10795668 (10p14), we found an interacting SNP rs367615 (5q21) with replication p = 0.01 and combined p = 4.19×10^−8^. Among the top marginal SNPs after LD pruning (n = 163), we identified an interaction between rs1571218 (20p12.3) and rs10879357 (12q21.1) (nominal combined p = 2.51×10^−6^; Bonferroni adjusted p = 0.03). Our study represents the first comprehensive search for GxG in CRC, and our results may provide new insight into the genetic etiology of CRC.

## Introduction

Genome-wide association studies (GWAS) have successfully identified single-nucleotide polymorphisms (SNPs) associated with colorectal cancer (CRC) [1]–[10]. As biologic candidates, those findings have enhanced our understanding of the genetic etiology of CRC. However, the susceptibility loci found so far explain only a small fraction of the genetic risk: the “missing heritability” problem [7]. Among other explanations, the lack of a comprehensive examination of gene-gene interaction (GxG) is often considered as one possible source for the unexplained heritability [11]–[14]. A recent paper also suggests that the missing heritability problem could be due to the overestimation of additive heritability if the assumption that there is no GxG or GxE interaction is incorrect [15]. The standard GWAS test for association is to use a single-locus approach, testing one SNP at a time across the entire genome; however, the underlying genetic mechanism of a complex disease, like CRC, probably involves interplays among multiple loci. Testing each locus individually without considering other loci with which it may interact may miss true genetic effects. Compared to the single-locus approach, there have been very few genome-wide examinations of GxG, probably at least partially due to the limited availability of individual-level large-scale GWAS data and analytical difficulties and limitations in computation given the massive number of possible interactions. A genome-wide study of psoriasis has reported compelling evidence for an interaction between variants at the *HLA-C* and *ERAP1* loci [16]. Another study identified a GxG between a previously identified locus *C1orf106 and* a new locus *TEC* for Crohn’s disease, with the interaction successfully replicated in an independent dataset [17]. So far, no GxG has been identified for CRC.

In this paper, we focus on testing pair-wise GxG for CRC using GWAS data in the Genetics and Epidemiology of Colorectal Cancer Consortium (GECCO) and the Colon Cancer Family Registry (CCFR) with a total sample size of 10,907 cases and 13,216 controls. We present a simple, but powerful method for testing interaction: the Average Risk Due to Interaction (ARDI). We performed a genome-wide search to identify SNPs interacting with previously identified CRC susceptibility loci in 14 independent regions (rs6687758/1q41, rs10936599/3q16.2, rs16892766/8q23.3, rs6983267/8q24, rs10795668/10p14, rs3802842/11q23, rs7136702/12q13.13, rs4444235/14q22.2, rs4779584/15q13, rs9929218/16q22.1, rs4939827/18q21, rs10411210/19q13, rs961253/20p12.3, rs4925386/20q13.33) [1]–[10]. We gave priority to these known susceptibility loci because they have been confirmed to be associated with CRC risk in previous studies. We also conducted a genome-wide search for pair-wise GxG. In order to alleviate the computational burden and reduce the number of multiple comparisons, we used marginal association screening and examined only pairwise interactions among the SNPs passing that screen.

## Results

### GxG for 14 known CRC Susceptibility Loci

After applying the QC and selection criteria, there were a total of 2,011,668 SNPs in common among studies in the Phase I studies (Materials and Methods; Table 1).

**Table 1:** Studies in Genetics and Epidemiology Colorectal Cancer Consortium (GECCO).

Study	Case†		Control†	Female				Colon				Age (yrs)				
				No.		%		No.			%	Mean			Range	
**Phase I**	N = 8,380	N = 10,558														
ASTERISK	948		947	782	41.3			661		69.7		65.3		40–99		
CCFR	1,171		983	1,077	50.0			569		48.6		56.2		19–83		
Colo2&3	87		125	95	44.8			59		67.8		65.2		38–86		
DACHS I	1,710		1,708	1,395	40.8			1,037		60.6		68.6		33–98		
DALS I	706		710	615	43.4			702		99.4		65.0		30–79		
MEC	328		346	313	46.4			241		73.5		63.0		45–76		
OFCCR^a^	650		522	610	52.0			435		66.9		62.1		29–77		
PLCO ¶	1,019		2,391	1,050	30.8			836		82.0		64.0		55–75		
VITAL	285		288	273	47.6			215		75.4		66.5		50–76		
WHI	1,476		2,538	4,014	100			1,157		78.4		67.9		50–79		
**Phase II**	N = 2,527	N = 2,658														
DACHS II	675		498	440	37.5		375		55.6			69.1	35–99			
DALS II	410		464	414	47.4		410		100			65.4	30–79			
HPFS	227		230	0	0		158		69.6			66.4	48–82			
NHS	553		955	1,508	100		420		75.9			59.8	44–69			
PHS	382		389	0	0		296		77.5			59.6	40–85			
PMH	280		122	402	100		206		73.6			64.5	50–75			
	Total = 10,907		Total = 13,216													
Adenoma studies	N = 826		N = 923													
HPFS Adv Adnm	313		345	0	0		245*		78.3			60.7	48–81			
NHS Adv Adnm	513		578	1,091	100		401*		78.1			57.0	44–69			

a Sample size excludes overlap with CCFR;

† Sample sizes given only for subjects clustering with HapMap CEU population in PCA (for data that has undergone QC);

¶ Includes participants with data downloaded from dbGaP prostate and lung studies;*for adenoma, number and % colon does not include subjects with adenomas located in both colon and rectum.

We selected interactions that have fixed-effect meta-analysis p-values <10^−6^ in Phase I for replication in Phase II. These interactions are summarized in Table 2. For SNPs that are in LD (r^2^>0.8), we reported only the most significant interacting SNP. Overall we identified 12 interactions with p<10^−6^ in Phase 1, including three interacting SNPs selected for each of the known loci rs6687758, rs4925386; two interacting SNPs selected for known locus rs7136702, and one interacting SNP for each of known locus rs4779584, rs10795668, rs9929218, and rs961253, respectively.

**Table 2:** Results for selected top interactions for known CRC loci with p-value less than 10^−6^ in Phase I studies.

Known Locus/region	Interacting SNP/region	MAF	Phase I InteractionOR (95% CI) P	Phase IIInteractionOR (95% CI) P	CombinedInteractionOR (95% CI) P	CombinedP_het_ *
rs6687758/1q41	rs9365723/6q25.3	0.43	0.75 (0.67–0.84) 5.83×10^−7^	0.95 (0.77–1.17) 0.63	0.79 (0.71–0.87) 3.79×10^−6^	0.75
	rs39453/7p15.3	0.37	0.74 (0.66–0.83) 6.34×10^−7^	0.92 (0.74–1.13) 0.42	0.78 (0.70–0.86) 2.08×10^−6^	0.54
	rs17777943/10q24.32	0.10	0.62 (0.51–0.74) 2.77×10^−7^	1.11 (0.80–1.55) 0.53	0.71 (0.60–0.83) 2.81×10^−5^	0.40
**rs10795668/10p14**	**rs367615/5q21.3**	**0.27**	**0.74 (0.66–0.84) 8.95×10** ^−**7**^	**0.76 (0.61–0.95) 0.01**	**0.74 (0.67–0.83) 4.19×10** ^−**8**^	**0.39**
rs7136702/12q13.13	rs17730929/4q13.2	0.10	0.62(0.51–0.74) 1.72×10^−7^	0.97 (0.68 –1.40) 0.88	0.68 (0.58–0.80) 2.78×10^−6^	0.09
	rs751147/14q21.2	0.27	0.73 (0.65–0.82) 8.89×10^−8^	1.16 (0.93–1.45) 0.19	0.80 (0.72–0.89) 3.5×10^−5^	0.13
rs4779584/15q13/*CRAC1*	rs10114408/9q22.31	0.24	0.67 (0.58–0.78) 3.26×10^−7^	0.93 (0.71–1.20) 0.56	0.73 (0.64–0.83) 2.54×10^−6^	0.28
rs9929218/16q22.1/*CDH1*	rs468905/16q21	0.28	0.76 (0.68–0.85) 7.14×10^−7^	1.03 (0.85 –1.26) 0.75	0.82 (0.74–0.90) 2.8×10^−5^	0.36
rs961253/20p12.3/*BMP2*	rs1661409/11q22.1	0.41	1.36 (1.20–1.54) 9.37×10^−7^	0.97 (0.78–1.20) 0.74	1.25 (1.12–1.39) 4.37×10^−5^	0.05
rs4925386/20q13.33	rs2500295/1p36.32	0.20	1.33 (1.19–1.50) 8.59×10^−7^	0.90 (0.73–1.11) 0.33	1.22 (1.10–1.35) 1.25×10^−4^	0.06
	rs4591517/3p24.3	0.28	1.31 (1.18–1.46) 6.92×10^−7^	1.06 (0.88–1.29) 0.54	1.25 (1.14–1.37) 3.26×10^−6^	0.21
	rs1394349/18q21.2	0.10	1.51 (1.28–1.78) 8.31×10^−7^	0.98 (0.72–1.34) 0.89	1.38 (1.19–1.59) 1.72×10^−5^	0.08

* P_het_ is the heterogeneity p-value.

**Figure 1:**
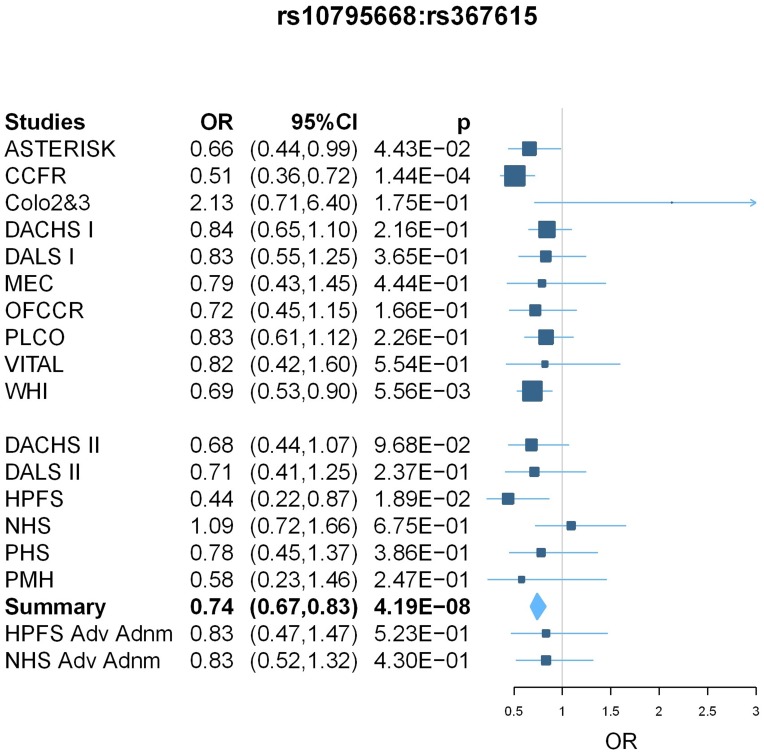
Forest plot for meta-analysis results of GxG between rs10795668 and rs367615. Box sizes are proportional to the inverse variance for each study and the lines depict the confidence intervals. The diamonds represent the fixed effects meta-analysis results, with the width of the diamond representing the confidence interval. The results of two advanced adenoma studies (HPFS Adv Adnm and NHS Adv Adnm) are shown at the bottom but not incorporated in the meta-analysis.

**Figure 2:**
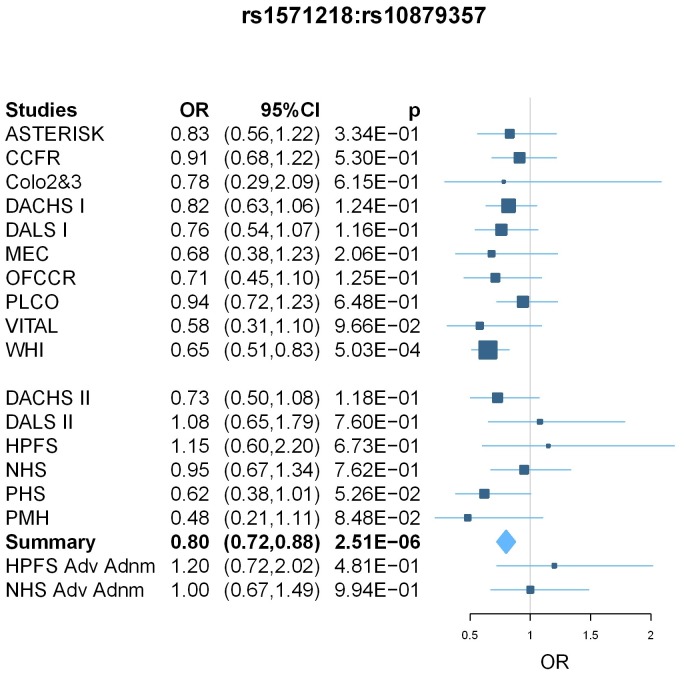
Regional interaction association plot for interacting region 5q21 with known CRC locus rs10795668. The left y-axis shows the -log10 of the meta-analysis interaction p value. The right y-axis shows the recombination rate. Each dot on the plot represents the result for one SNP. The diamond dot represents SNP rs367615 and the round dots represent other SNPs. Difference colors of SNPs indicate different LD strength between the corresponding SNP and rs367615, measured by r^2^. The bottom of the figure shows the genes in the plotted region.

Within Phase II, the interaction between known loci rs10795668 and rs367615 showed evidence for replication (OR = 0.76, 95% CI 0.61–0.95; p = 0.01) with a combined Phase I and II OR of 0.74 (95%CI 0.67–0.83; p = 4.19×10̂-8). rs367615 is located on 5q21 and has a MAF of 0.22 in CEU population. Additional inclusion of two advanced colorectal adenoma studies in the replication study further strengthened the statistical significance level of the replication (OR = 0.78 and 8.97×10^−3^); OR and p-value for Phase I, II and advanced adenoma studies combined are 0.75 and 2.88×10^−8^. rs10795668 was genotyped in 10 studies and imputed in 11 studies with average imputation R^2^ of 0.97 (range from 0.92 to 1.00); rs367615 was genotyped in 4 studies and imputed in 17 studies with average R^2^ of 0.98 (range from 0.91 to 1.00). The forest plot showing individual study results is presented in Figure 1. We did not observe evidence for heterogeneity, and random effects results are similar to fixed effects results for this interaction. Figure 2 shows the regional association plot. Several LD partners of rs367615 also show evidence of interaction with rs10795668.

We also examined the two-locus interaction pattern for the SNP pair described above using a unrestricted model. Table 3(a) summarizes the OR and sample size for each genotype combination relative to the reference genotypes for Phase I and II studies combined. Table 3(b) and Table 3(c) summarize the OR for each SNP stratified by the genotypes of the other. In Table 3, we can see that subjects who carry AG genotype for rs10795668 and CT genotypes for rs367615 have a statistically significantly increased disease risk compared to those who carry reference genotypes at both loci (rs10795668:GG/rs367615:TT). However, for subjects who carry AG or AA genotype for rs10795668, carrying CT genotypes significantly decreases the disease risk. The interaction OR can also be calculated from the table. For example, if there were no interaction effect, samples that carry GG for rs10795668 and CT for rs367615 would have an increased risk compared to the reference group (OR would be 1.03*1.11 = 1.14). However, they actually have a statistically significantly decreased risk (OR = 0.87; p = 2.99×10^−3^) because of the interaction (OR = 0.76). The interaction OR’s of rs10795668:AG/rs367615:CT, rs10795668:AG/rs367615:CC, rs10795668:AA/rs367615:CT and rs10795668:AA/rs367615:CC in Table 3(a) can easily be calculated to be 0.76, 1.01, 0.60 and 0.89, respectively. This looks like an unusual interaction pattern. However, it is worth noting that the sample size is relatively small when the genotype of rs367615 is CC and as a result, all OR estimates in the third column have large p-values and wide confidence intervals. To account for the small sample size, and to aid interpretation, we re-constructed the interaction table by combining the CT and CC genotype of rs367615 and the AG and AA genotypes of rs10795668. Table 3(d) shows that the CT/CC genotypes of rs367615 have an increased risk when the genotype of rs10795668 is GG. On the other hand, the combination of AG/AA genotype of rs10795668 and CT/CC genotype of rs367615 has a protective effect.

**Table 3:** Interaction pattern between rs10795668 and rs367615.

Table 3(a).			
	rs367615:TT	rs367615:CT	rs367615:CC
rs10795668:GG	1	1.11 (1.02–1.22) 1.73×10^−2^	1.07 (0.87–1.32) 5.18×10^−1^
	3212/3968	1599/1783	229/246
rs10795668:AG	1.03 (0.95–1.10) 5.00×10^−1^	0.87 (0.80–0.95) 2.99×10^−3^	1.12 (0.92–1.36) 2.50×10^−1^
	2896/3585	1355/1858	349/316
rs10795668:AA	1.14 (1.01–1.30) 3.19×10^−2^	0.76 (0.64–0.89) 9.35×10^−4^	1.10 (0.85–1.42) 4.52×10^−1^
	691/771	319/493	250/193
For each combination of genotypes, we computed the odds ratio (95% CI), and pvalue relative to the baseline group (rs10795668:GG; rs367615:TT). We also list the sample size for cases/controls.			
**Table 3(b)** **. OR of rs367615 stratified by rs10795668**			
	**rs367615:TT**	**rs367615:CT**	**rs367615:CC**
rs10795668:GG	1	1.11 (1.02–1.22) 1.73×10^−2^	1.07 (0.87–1.32) 5.18×10^−1^
rs10795668:AG	1	0.85 (0.78–0.93) 5.82×10^−4^	1.09 (0.90–1.33) 3.70×10^−1^
rs10795668:AA	1	0.66 (0.55–0.80) 2.96×10^−5^	0.96 (0.73–1.27) 7.88×10^−1^
**Table 3(c)** **. OR of rs107895668 stratified by rs367615**			
	**rs367615:TT**	**rs367615:CT**	**rs367615:CC**
rs10795668:GG	1	1	1
rs10795668:AG	1.03 (0.95–1.10) 5.00×10^−1^	0.78 (0.70–0.87) 5.27×10^−6^	1.05 (0.80–1.37) 7.43×10^−1^
rs10795668:AA	1.14 (1.01–1.30) 3.19×10^−2^	0.68 (0.57–0.81) 1.17×10^−5^	1.03 (0.76–1.40) 8.58×10^−1^
**Table 3(d)** **. Interaction pattern between rs10795668 and rs367615 by combining the heterozygous and homozygous minor genotypes.**			
	**rs367615:TT**	**rs367615:CT/CC**	
rs10795668:GG	1	1.11 (1.02–1.20) 1.96×10^−2^	
rs10795668:AG/AA	1.05 (0.98–1.12) 1.92×10^−1^	0.88 (0.81–0.95) 1.67×10^−3^	

As we have fit ARDI and unrestricted model for the top interaction between rs10795668 and rs367615, it would be interesting to also see the results from the multiplicative model. The multiplicative interaction OR is estimated to be 0.83 with combined p = 3.14×10^−6^, which is less significant compared to ARDI model.

### GxG among Top Marginal SNPs

Based on the meta-analysis results of the marginal association analysis for all except two advanced adenoma studies, we selected 606 SNPs for testing GxG with MAF>0.05, average R^2^>0.3, and both fixed and random effect meta-analysis p<0.0001. Both fixed and random effect p-values were used because we wanted to avoid selecting SNPs with signal dominated by a few studies. With this selection criterion, all chosen SNPs had heterogeneity p-value >0.1. After applying a LD-pruning routine (Materials and Methods), 163 SNPs remained.

**Figure 3:**
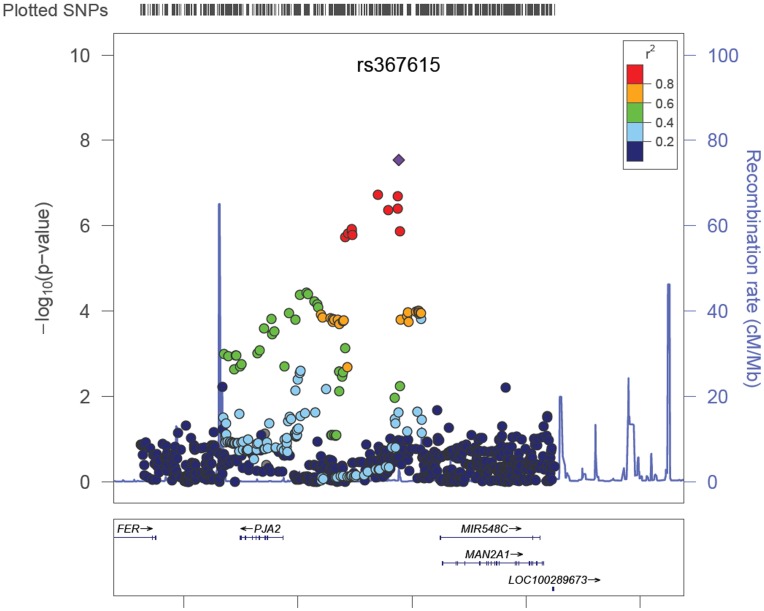
Forest plot for meta-analysis results of GxG between rs1571218 and rs10879357. Box sizes are proportional to the inverse variance for each study and the lines depict the confidence intervals. The diamonds represent the fixed effects meta-analysis results, with the width of the diamond representing the confidence interval. The results of two advanced adenoma studies (HPFS Adv Adnm and NHS Adv Adnm) are shown at the bottom but not incorporated in the meta-analysis.

In Phase I, we observed five pairs of SNPs with fixed-effect meta-analysis interaction p-value<5×10^−5^ (Table 4). These five interactions point to 3 independent findings, as indicated by correlation of the first two SNPs (rs2170568 and rs7006896, r^2^ = 0.78) and the next two SNPs (rs2200579 and rs10879357, r^2^ = 0.75). In the replication, the GxG between rs1571218/20p12.3 and the two correlated SNPs rs2200579 and rs10879357 which are on 12q21.1 are significant at level 0.1 (p-values are 0.04 and 0.06, respectively), with interaction ORs in the same direction. The combined Phase I and II analysis OR and p-values are 0.81 and 4.61×10^−6^ and 0.80 and 2.51×10^−6^, respectively. The interaction between rs1571218 and rs10879357 passed the Bonferroni correction with threshold 3.79×10^−6^ = 0.05/(163*162/2). After including the two advanced colorectal adenoma studies, the replication OR and p-value are 0.89 and 0.17 for rs1571218 and rs10879357; the combined analysis OR and p-value are 0.82 and 1.15×10^−5^. rs1571218 was well imputed in all studies with average imputation R^2^ of 0.95 (range from 0.91 to 0.98); rs10879357 was genotyped in 11 studies and imputed in 10 studies with average R^2^ of 0.78 (range from 0.76 to 0.80). The forest plot shows consistent results across the individual studies (Figure 3). Again, we did not observe heterogeneity and random effects results are similar to fixed effects results.

**Table 4:** Results for selected top interactions among top marginal loci with p-value less than 5×10^−5^ in Phase I studies.

Interacting SNP 1/region	MAF 1	Interacting SNP 2/region	MAF 2	Phase I InteractionOR(95% CI) P	Phase II InteractionOR(95% CI) P	Combined InteractionOR(95% CI) P	Combined P_het_
rs11106204/12q21.33	0.21	rs2170568/8q24.21	0.17	1.38 (1.19–1.60) 1.33×10^−5^	0.97 (0.75–1.26) 0.82	1.27 (1.12–1.44) 2.24×10^−4^	0.02
		rs7006896/8q24.21	0.17	1.38 (1.19–1.59) 1.29×10^−5^	0.97 (0.75–1.26) 0.82	1.27 (1.12–1.44) 2.16×10^−4^	0.03
**rs1571218/20p12.3**	**0.48**	**rs2200579/12q21.1**	**0.31**	**0.81 (0.73–0.89) 4.72**×**10** ^−**5**^	**0.81 (0.67–0.99) 0.04**	**0.81 (0.74–0.89) 4.61**×**10** ^−**6**^	**0.77**
		**rs10879357/12q21.1**	**0.38**	**0.79 (0.70–0.88) 1.37**×**10** ^−**5**^	**0.83 (0.69–1.01) 0.06**	**0.80 (0.72–0.88) 2.51**×**10** ^−**6**^	**0.74**
rs4766549/12q24.11	0.16	rs10879357/12q21.1	0.38	0.73 (0.63–0.85) 3.03×10^−5^	0.90 (0.70–1.17) 0.43	0.77 (0.68–0.88) 6.3×10^−5^	0.71

P_het_ is the heterogeneity p-value.

The two-locus interaction pattern for rs1571218 and rs10879357 is summarized in Table 5(a). The OR for each SNP stratified by the genotypes of the other are summarized in Table 5(b) and Table 5(c). In Table 5, we can see that all non-reference combinations are associated with an increased disease risk compared to the reference group. However, due to interactions with inverse associations, the risks are not as large as they would have been without interaction. For example, if there were no interaction effect, persons who carry AG for rs10879357 and GT for rs1571218 would have an higher risk compared to the reference group (OR = 1.12×1.18 = 1.32). However, the risk is lower (OR = 1.08) because of the interaction (OR = 0.82). Computed as above, the interaction OR’s of rs1571218:GT/rs10879357:AG, rs1571218:GT/rs10879357:AA, rs1571218:TT/rs10879357:AG and rs1571218:TT/rs10879357:AA in Table 5(a) are 0.82, 0.84, 0.83 and 0.89, respectively, which seems to follow a dominant genetic model. Table 5(b) shows the deleterious association with allele A of rs10879357 seems to be offset by the allele T of rs1571218. A similar pattern can also be observed for rs1571218 in Table 5(c). This indicates that there may be an exclusive interaction between rs10879357 and rs1571218.

**Table 5:** Interaction pattern between rs1571218 and rs10879357.

Table 5(a)			
	rs10879357:GG	rs10879357:AG	rs10879357:AA
rs1571218:GG	1	1.12 (1.00–1.25) 4.96×10^−2^	1.36 (1.18–1.57) 2.22×10^−5^
	1383/1918	1930/2457	729/781
rs1571218:GT	1.18 (1.06–1.31) 3.31×10^−3^	1.08 (0.98–1.20) 1.32×10^−1^	1.35 (1.18–1.54) 1.03×10^−5^
	1781/2186	2384/3022	948/1008
rs1571218:TT	1.35 (1.16–1.57) 9.59×10^−5^	1.25 (1.09–1.44) 1.35×10^−3^	1.63 (1.33–2.00) 2.20×10^−6^
	599/649	787/885	345/308
For each combination of genotypes, we computed the odds ratio (95% CI) and pvalue relative to the baseline group (rs1571218:GG; rs10879357:GG). We also list the sample size for cases/controls.			
**Table 5(b)** **. OR of rs10879357 stratified by rs1571218**			
	**rs10879357:GG**	**rs10879357:AG**	**rs10879357:AA**
rs1571218:GG	1	1.12 (1.00–1.25) 4.96×10^−2^	1.36 (1.18–1.57) 2.22×10^−5^
rs1571218:GT	1	0.92 (0.83–1.01) 8.81×10^−2^	1.14 (1.01–1.30) 4.05×10^−2^
rs1571218:TT	1	0.93 (0.78–1.11) 4.10×10^−1^	1.21 (0.96–1.51) 1.03×10^−1^
**Table 5(c)** **. OR of rs 1571218 stratified by rs10879357**			
	**rs10879357:GG**	**rs10879357:AG**	**rs10879357:AA**
rs1571218:GG	1	1	1
rs1571218:GT	1.18 (1.06–1.31) 3.31×10^−3^	0.97 (0.88–1.07) 5.06×10^−1^	0.99 (0.84–1.16) 8.85×10^−1^
rs1571218:TT	1.35 (1.16–1.57) 9.59×10^−5^	1.12 (0.98–1.28) 8.69×10^−2^	1.20 (0.96–1.49) 1.12×10^−1^

We also calculated the multiplicative interaction OR ( = 0.94) and combined p ( = 0.08) between rs1571218 and rs10879357.

## Discussion

In this large study, we performed a genome-wide search for pairwise GxG for each of the known CRC susceptibility loci and among top SNPs with small p-values for marginal effects. To our knowledge, this represents the first comprehensive GxG scan for colorectal cancer. The most significant interaction found in our examination of known loci and other SNPs genome-wide was between the known locus rs10795668 (10p14) and rs367615 (5q21) with replication p = 0.01 and combined p = 4.19×10^−8^. The effect sizes are very similar in Phase I and Phase II studies, and there is no evidence of heterogeneity (P_het_ = 0.39). Among the top marginal SNPs, the most promising interaction was between rs1571218 (20p12.3) and rs10879357 (12q21.1) (nominal p = 2.51×10^−6^; adjusted p = 0.03). Again, the effect sizes are very similar in Phase I and Phase II studies and there is little evidence for heterogeneity (P_het_ = 0.74).

The known locus rs10795668 in our identified interaction is located in an intergenic region within 10p14. So far, the function of this SNP has not been clearly defined and it has not been related to specific gene(s). The nearest predicted genes in this region are *BC031880* and *HV455515* and *DD431424*, the latter two are newly identified regulator genes for *hTERT*, a genetic region that contains susceptibility loci of multiple different cancers, including colorectal cancer [9], [18]–[27]. Other genes close by are *TAF3* and *GATA3* (∼0.6 M bp). *GATA3* belongs to the GATA family of transcription factors, which are important for T-cell development. *TAF3* is a *TBP*-associated factor (TAF); these contribute to promoter recognition and selectivity and act as antiapoptotic factors [28]. rs10795668 has also been found to be correlated with the expression of *ATP5C1* [29], which is involved in cell metabolism. rs367615 is located in an intergenic region within 5q21, where there is one member of the Wnt signaling pathway (*APC*) known to be important in both familial and non-familial colorectal cancer as well as *MCC*, perhaps also important in CRC [30], [31]. The closest genes to rs367615 are *PJA2*, *MAN2A1* and *FER*. *PJA2* is responsible for ubiquitination of cAMP-dependent protein kinase type I and type II-alpha/beta regulatory subunits and for targeting them for proteasomal degradation [32]. *PJA2* has been found to bind the ubiquitin-conjugating enzyme *UbcH5B* [33], which functions in the ubiquitination of the tumor-suppressor protein p53. *FER* regulates cell-cell adhesion and mediates signaling from the cell surface to the cytoskeleton via growth factor receptors. *MAN2A1* is a Golgi enzyme important in N-glycan processing [34]. Upon additional bioinformatic analysis, we identified two potential functional candidates, rs2201016 and rs2201015, that are in strong LD with rs367615 (r^2^ values of 1 and 0.916 respectively). As shown in the UCSC Genome Browser view (Figure S2, Table S2), rs2201016 and rs2201015 fall within a region of strong DNAse hypersensitivity and evolutionary conservation. As shown in Table 3(a), the interaction seems to be driven by the CT group of rs367615, which is an uncommon phenomenon and may be related to heterozygote advantage. However, the minor allele heterozygous (CC) genotype is relatively rare, making it difficult to conclusively estimate the effect size in that genotyped. Although both SNPs point to potentially relevant genes involved in cancer development, advancing basic research and translating these GWAS findings in to clinical benefit will require further functional characterization through *in vitro* and *in vivo* analysis.

We observed a statistically significant interaction between rs1571218/20p12.3 and rs10879357/12q21.1 (and a marginally significant interaction with a close by and correlated SNP, rs2200579). The SNP rs1571218 is in the same region (20p12.3) and modestly correlated (r^2^ = 0.56) with the known CRC locus rs961253. The closest gene is bone morphogenetic protein 2 (*BMP2*), which is part of transforming growth factor-beta (TGF-β) pathway. The TGF-β pathway plays an important role in cell proliferation, differentiation, and apoptosis [35] and is established as important in CRC [36]. Two interacting SNPs rs2200579 and rs10879357 are close together (<4 k bp apart) at 12q21.1 and are correlated (r^2^ = 0.76). These SNPs fall in the intronic region of *TPH2*, which is a rate-limiting enzyme in the synthesis of serotonin [37]. Serotonin is known to be involved in numerous central nervous activities. There is also evidence that serotonin is mitogenic in different cancer cell lines [38]–[40]. One study has shown that lack of serotonin causes a reduction of tumor growth in a mouse model of colon cancer allografts [41]. Further bioinformatic analysis revealed that rs10879357 is in LD (r2 = 0.697) with a synonymous coding SNP (rs4290270) in the exonic region towards the tail end of TPH2. Further *in vivo* or *in vitro* analysis is necessary to determine whether this variant has a functional impact such as mRNA stability. Because rs2200579 and rs10879357 are in a gene rich region, it is also possible that the SNPs impact genes other than *TPH2*.

In this paper, studies were divided into Phase I and II according to the time their genotype data became available. Phase II was expected to serve as validation/replication of Phase I. For the known loci GxG search, the Phase II p-value between rs10795668 and rs367615 is 0.01, which is nominally significant at the 0.05 level but does not pass the Bonferroni threshold (0.05/12). Among the top marginal SNPs, the Phase II p-value between rs1571218 and rs10879357 also does not pass the Bonferroni threshold (0.05/5) even when the combined p-value passes the Bonferroni threshold (3.79×10^−6^ = 0.05/(163*162/2)). In fact, combined test was recommended in two-stage GWAS because the replication test has been shown to be less efficient compared to combined test [42]. Therefore, larger sample size is needed to reach enough power to replicate our findings.

Adenomas are well known precursor lesions of colorectal cancer. Accordingly, we investigated if the observed interactions for colorectal cancer are also seen in advanced colorectal adenomas. Our findings suggest that the interaction between rs10795668 and rs367615 is present in advanced adenomas, suggesting that the genetic variants may act early in the development of colorectal cancer. In contrast, the interaction between rs1571218 and rs10879357 was not observed in advanced adenoma, which may suggest that the genetic variants act at a later stage of cancer development. However, the findings need to be interpreted with caution, as the number of adenomas is relatively small (<1000 cases).

In marginal association analysis, the most commonly used model is the log-additive model, where the genotype is coded as 0, 1 or 2 (based on the number of count alleles). It is therefore natural to use the same genetic coding in a two-locus interaction model to test for GxG. In the interaction model, the interaction effect is modeled by the product of the genotypes of two SNPs. As we can see in Table 6(a), this interaction model assumes that the interaction when both SNPs have homozygous genotype ( = 2) is four times as large as when both SNPs have heterozygous genotype ( = 1). In other words, this model assumes 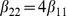 in the Table 6(b), which is a strong assumption. Indeed, we can see that the interaction pattern in Table 3(a) is not consistent with this assumption. Some simple calculations demonstrate that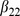 = log(0.89), which actually represents a smaller effect size compared to 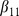 = log(0.76). In fact, we have found in simulation that violation of this assumption can result in substantial loss of power (Figure S1). A cautious way to avoid posing such a strong assumption is to use an unrestricted model, which is also a widely adopted method [17], [43]. Using an unrestricted model can avoid violation of assumptions but may result in substantial loss of power because of the increased degrees of freedom (from 1 to 4). Our ADRI method uses the same genetic coding as the log-additive model to allow allelic effects for main effects, which also makes the interaction test independent of the marginal screening. For the interaction, our method estimates the average interaction effect 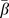 of 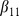, 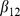, 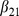, and 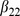. Because 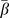 is an average effect, it is less prone to heterogeneity among studies. As a result, our method is more stable and reproducible compared to the unrestricted and log-additive model. It is worth pointing out that when the underlying genetic model is indeed log-additive, ARDI is less powerful compared to the regular interaction model with log-additive genetic coding. For future applications, a model selection technique needs to be developed to determine the most appropriate model with the least loss of power. Another worth noting point is that the case-only model, which assumes independence between SNPs in controls, is known to be more powerful than the case control combined model while testing for gene-gene interaction [44], [45]. In our case, ARDI is a case control combined approach so the power can also be boosted by using its case-only counterpart. We did not implement the case-only ARDI for two reasons: it is relatively hard to completely avoid violation of the independence assumption (thus maintain the type I error rate) in case-only model due to the complexity of the LD structure of the human genome, i.e, long-range LD [46]; in addition, the current available package [47] for fitting a case-only model with covariates are only applicable to genotyped SNPs while our data include imputed dosages. As an on-going work, we are developing a package that can fit case-only model for two imputed SNPs while adjusting for covariates.

**Table 6:** An illustration of different two-SNP interaction models. SNP 1 has genotype AA, Aa and aa; SNP 2 has genotype BB, Bb, and bb. A and B are the major alleles for SNP1 and 2, respectively.

(a)			
	AA	Aa	aa
BB	1	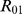	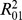
Bb	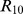	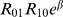	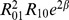
bb	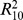	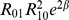	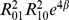
**_(b)_**			
	**AA**	**Aa**	**aa**
BB	1	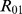	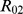
Bb	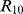	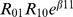	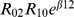
bb	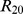	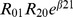	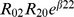
**_(c)_**			
	**AA**	**Aa**	**aa**
BB	1	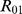	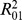
Bb	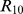	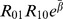	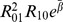
bb	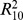	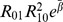	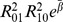

Each entry in the tables represents the risk of the corresponding genotype combination relative to the baseline (AA/BB). (a) Multiplicative interaction model; (b) Unrestricted interaction model; (c) Average Risk Due to Interaction (ARDI) model.

GxG is usually defined as the departure from main effects [13]. Therefore, if the underlying main effects are not correctly specified, the residual main effects could be incorporated as part of the interaction effect in the statistical model [48]. As a result, testing interaction implicitly evaluates the residual main effect and interaction effect jointly. We keep the main effects as log-additive in ARDI, mainly because we want to be consistent with the usual log-additive model used in the marginal association analysis so that ARDI test is independent of the marginal screening. However, the log-additive main effect is prone to model misspecification. We observed this in our study for four of the known loci, rs10936599, rs6983267, rs4779584 and rs961253. These SNPs all showed an inflated 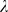
for the interaction tests when using additive genetic coding for the main effect. In all four cases, the inflation 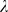
diminished after we switched to unrestricted coding with no misspecification. VanderWeele and Laird (2011) used a similar approach to protect against potential misspecification of main effects [49]. We tried ARDI with unrestricted main effect on our top findings. Under the ARDI model with unrestricted main effect, the interaction between known locus rs10795668 and rs367615 has an OR of 0.75 and combined p = 1.07×10^−6^ (original OR = 0.74 and combined p = 4.19×10^−8^); interaction between rs1571218 and rs10879357 has an OR of 0.83 and combined p = 3.90×10^−4^ (original OR = 0.80 and combined p = 2.51×10^−6^). As we can see, the OR’s stay largely the same and there are still strong signals of interaction. However, the p-values get larger in the new model, which could be due to random fluctuations between different models, or also could be a sign of main effect misspecification. Hence, our interaction test results should be interpreted with caution.

In our GxG search, we performed genome-wide interaction search for each known CRC locus and all other SNPs, including the SNPs that are in LD with it. This raises an important concern whether it is appropriate to test GxG between two SNPs that are in high LD. As an alternative, it is of interest to conduct haplotype analysis on the nearby regions of the known loci. We also prioritized SNPs based on their marginal association strength, using established methods [50]. Our reasoning is that if a SNP is involved in GxG, it is also likely that it will show evidence of some marginal effect. As most SNPs in GWAS are null, selecting a subset of SNPs that are more likely to show interaction could increase the power substantially as it reduces the overall multiple comparison burden. However, it is also possible for a SNP to show little or no marginal association if it is involved in interaction that is in the opposite direction to that seen with the main effect. In this case, we would not be able to find those qualitative interactions using our screening. Future research is needed to explore methods to complement the marginal association screening while still restricting the number of tests at a reasonable level to ensure reasonable power.

In this paper, we focused on pair-wise interactions. For higher order interactions, data mining methods such Random Forest [51], [52] and Multifactor Dimensionality Reduction [53] are preferred compared to the traditional regression-based methods because of the scarcity of the potential high-order contingency table [13]. As pointed out by Cordell [13], most of the high-order data mining methods, except for Random Forest, are computationally intensive, and hence, are not easily applicable to GWAS data. In addition, as the data mining methods are nonparametric, permutation tests are usually needed to produce p-value. Unfortunately they are generally computationally impossible for GWAS. Given the aforementioned limitations, one possible practical approach for searching for higher order GxG is to use Random Forest in a discovery dataset and use traditional regression-based methods to replicate the findings.

It is important to note that we focused on testing statistical interaction in this paper and statistical interaction does not always warrant a biologic or mechanistic interaction [54]. Mechanistic interaction can be tested using the sufficient cause framework [55], which is out of the scope of this paper.

In summary, our study is the first to comprehensively search for GxG for CRC. We have found evidence for two interactions associated with CRC risk. Further studies are needed to evaluate these interactions and to study the underlying molecular mechanisms.

## Materials and Methods

### Study Participants

The studies used in this analysis, including number of cases and controls, are listed in Table 1, with each study described in detail in the Text S1. In brief, colorectal cancer cases were defined as adenocarcinoma of colon and rectum (International Classification of Disease Code 153–154) and were confirmed by medical record, pathology report, or death certificate. Advanced colorectal adenoma cases are defined as adenoma ≥1 cm in diameter and/or with tubulovillous, villous, or high-grade dysplasia/carcinoma-in-situ histology, and were confirmed by medical record, histopathology, or pathology report. All participants provided written informed consent and studies were approved by the Institutional Review Board.

### Genotyping

We conducted genome-wide scans for all studies. GECCO GWAS consisted of participants of European ancestry within 13 studies including the French Association Study Evaluating RISK for sporadic colorectal cancer (ASTERISK); Hawaii Colorectal Cancer Studies 2 and 3 (Colo2&3); Darmkrebs: Chancen der Verhutung durch Screening (DACHS); Diet, Activity, and Lifestyle Study (DALS); Health Professionals Follow-up Study (HPFS); Multiethnic Cohort (MEC); Nurses’ Health Study (NHS); Ontario Familial Colorectal Cancer Registry (OFCCR); Physician’s Health Study (PHS); Postmenopausal Hormone study (PMH); Prostate, Lung, Colorectal Cancer, and Ovarian Cancer Screening Trial (PLCO); VITamins And Lifestyle (VITAL); and the Women’s Health Initiative (WHI). Phase one genotyping on a total of 1,709 colon cancer cases and 4,214 controls from PLCO, WHI, and DALS (PLCO Set 1, WHI Set 1, and DALS Set 1) was done using Illumina Human Hap 550 K, 610 K, or combined Illumina 300 K and 240 K, and has been described previously [9]. A total of 650 colorectal cancer cases and 522 controls from OFCCR are included in GECCO from previous genotyping using Affymetrix platforms [2]. A total of 5,540 colorectal cancer cases and 5,425 controls from ASTERISK, Colo2&3, DACHS Set 1, DALS Set 2, MEC, PMH, PLCO Set 2, VITAL, and WHI Set 2 were successfully genotyped using Illumina HumanCytoSNP. A total of 1,837 colorectal cancer cases and 2,072 controls from HPFS, NHS, PHS, and DACHS set 2, as well as a total of 826 advanced adenoma cases and 923 controls from HPFS and NHS were successfully genotyped using Illumina HumanOmniExpress. A population-based case-control GWAS from CCFR (1,171 cases and 983 controls) was successfully genotyped using Illumina Human1M or Human1M-Duo [56].

We divided the studies into two phases according to the time their genotype data became available (Table 1). We used the Phase I studies (10 studies; 8,380 cases and 10,558 controls) as the discovery set and Phase II studies (6 studies; 2,527 cases and 2,628 controls) as the replication set. In addition, there are two advanced colorectal adenoma studies, which we use to evaluate whether the interactions found for carcinoma are also associated with advanced adenoma.

DNA was extracted from blood samples or, in the case of a subset of DACHS, MEC, and PLCO samples, and all VITAL samples, from buccal cells using conventional methods. All studies included 1 to 6% blinded duplicates to monitor quality of the genotyping. All individual-level genotype data were managed centrally at University of Southern California (CCFR), the Ontario Institute for Cancer Research (OFCCR), the University of Washington (HPFS, NHS, PHS, and the second GWAS of DACHS), or the GECCO Coordinating Center at the Fred Hutchinson Cancer Research Center (all other studies) to ensure a consistent quality assurance and quality control approach and statistical analysis. Samples were excluded based on call rate, heterozygosity, unexpected duplicates, gender discrepancy, and unexpectedly high identity-by-descent or unexpected concordance (>65%) with another individual. All analyses were restricted to samples clustering with the CEU population in principal component analysis, including the three HapMap populations as a reference. SNPs were excluded if they were triallelic, not assigned an rs number, or were reported as not performing consistently across platforms. Additionally, they were excluded based on call rate (<98%), Hardy Weinberg Equilibrium in controls (HWE, p<10^−4^), and minor allele frequency. To place studies on a common set of autosomal SNPs, all studies were imputed to HapMap II release 24, with the exception of OFCCR, which was imputed to HapMap II release 22. CCFR was imputed using IMPUTE [57], OFCCR was imputed using BEAGLE [58], and all other studies were imputed using MACH [59]. Given the high agreement of imputation accuracy among MACH, IMPUTE, and BEAGLE [60] the common practice to use different imputation programs is unlikely to cause heterogeneity [61]. Imputed data were merged with genotype data such that genotype data were preferentially selected if a SNP had both types of data, unless there was a difference in terms of reference allele frequency (>0.1) or position (>100 base pairs), in which case imputed data were used. As a measurement of imputation accuracy, we calculated R^2^ [59].

For the GxG analysis, we restricted the search to SNPs with MAF>0.05 and imputation R^2^>0.3 because there is inadequate power to detect interactions between less frequent variants or variants with lower imputation quality given the current sample size.

### Statistical Method

#### GxG model

A logistic regression model was used to assess GxG for each SNP pair tested. In particular, we used a simple yet powerful approach named “Average Risk Due to Interaction (ARDI)” to test for GxG. In this approach, the main effects of the SNPs are log-additive and the interaction effect is the averaged deviation from the main effects. This is in contrast to the usual modeling of the interaction effect for log-additive model, where the interaction term is the product of the two SNPs. To see this, we consider two SNPs, G_1_ ( = AA, Aa or aa) and G_2_ ( = BB, Bb, or bb) while A and B are the major alleles for G_1_ and G_2_, respectively. Table 6(a) shows the usual interaction model with log-additive effects. Under this model, the interaction effect of aa/bb combination relative to the main effects is exp(4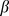), which is considerably larger than the Aa/Bb combination, which is exp(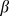). One way to avoid this strong assumption of interaction pattern is to use an unrestricted model (Table 6(b)), which models the interaction effect by four parameters 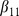, 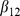, 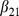, and 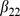. A four-degrees-of-freedom test is needed to test for the interaction effect, which may result in a substantial power loss. We therefore modeled the average interaction effect with one parameter 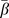 while keeping the main effect as log-additive (ARDI) (see Table 6(c)). This modeling avoids the strong assumption of the usual modeling of interactions with log-additive main effects, and yet gains power by having only one parameter to test for interaction. We keep the main effects as log-additive, mainly because we want to be consistent with the usual log-additive model used in the marginal association analysis. We have conducted extensive simulation studies to compare the performance of ARDI with multiplicative interaction model and unrestricted interaction model. Simulation results show that ARDI has favorable performance while the underlying interaction pattern is unknown (see Text S1, Table S1 and Figure S1). We have also tried both multiplicative model and ARDI in the Phase I studies and ARDI yielded more significant results genome widely, which supported the conclusion from the simulation because in this case the true underlying interaction is unknown and likely to vary among SNPs. Hence, we chose ARDI as our GxG model. Specifically, the ARDI model can be written as:



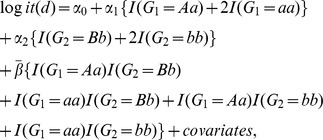
where d is the disease status (0/1), 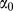 is the intercept, 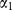 and 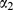 are the main effects, 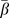 is the ARDI interaction effect. The hypothesis is to test whether 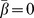. For all models, we adjusted for age, sex, study center, and the first three principal components from EIGENSTRAT [62] to account for population substructure.

#### GxG searching strategy

We performed genome-wide interaction testing for each of the 14 known CRC susceptibility loci and all other 2.1 M SNPs in the Phase I studies. SNPs with p<10^−6^ in Phase I were examined in the Phase II studies using the same ARDI model.

We also conducted a genome-wide search of GxG for all SNPs, using a two-stage approach. In the first stage, we did a genome-wide marginal association test with additive genetic coding for all 2.1 M SNPs. Then we selected SNPs with marginal association p-value <0.0001 for the second stage and searched for pair-wise interactions among the selected SNPs. We selected 0.0001 as the cutoff so that around 100 independent regions would be selected assuming there are one million independent regions genome-wide [63]. It has been shown that the screening on marginal association is independent of the GxG test as long as the genetic coding for the main effect is the same as in the marginal association testing [50]. Because both the marginal association test and the main effect of ARDI use additive genetic coding, we need to adjust only for the number of interaction tests performed in the second stage to maintain the correct type I error level.

We observed 606 SNPs with marginal association p<0.0001. However, the 606 selected SNPs are not independent due to linkage disequilibrium (LD) between SNPs. As a result, if we used the number of pair-wise interactions among those 606 SNPs (n = 183,315) with a Bonferroni correction to compute the adjusted alpha level, the result would be too conservative. Therefore, we performed a pruning based on LD. First, the selected SNPs were ranked based on the marginal association p-value. Starting with the first SNP (SNP with the strongest signal), we removed all SNPs that have a LD r^2^>0.8 with that SNP. Then we moved to the next SNP, and repeated the procedure until we reached the end of the list. A total of 163 SNPs remained after this LD pruning. We then tested for GxG among these SNPs in Phase I studies. Interactions with p<5×10^−5^ were selected for Phase II (so the expected number of false positive based on total 163*162/2 = 13,203 interaction tests is less than one).

### Meta-analysis

We used the fixed-effect meta-analysis to combine interaction estimates across studies. In this approach, we used the inverse-variance weighting to combine the regression coefficient estimates from each study. As previously demonstrated [64], the imputation quality is automatically incorporated into meta-analysis with the inverse-variance weighting. We report the summary estimate, standard error, and 95% confidence interval, as well as the heterogeneity p-value for the meta-analysis. For top findings we examined whether a random effects model would result in substantively different results from our fixed effects model. We also examined forest plots for top interaction findings. We present meta-analysis results for Phase I alone, Phase II alone, and Phase I and II combined.

### Genomic Inflation

We checked the QQ plot and genomic inflation factor 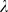 for the GxG meta-analysis results of each known locus. Among 14 known loci, 10 of them showed no systematic bias, with 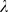 ‘s less than 1.05. However, rs10936599, rs6983267, rs4779584 and rs961253 showed some indication of an inflated 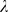 (1.10–1.78). For each of these SNPs we found that the systematic inflation was due to inappropriate additive genetic coding for the main effect. If the main effect for a SNP does not follow an additive model (with the heterozygote effect half way between the two homozygotes on the log scale), but additive coding is used, this misspecification results in a residual main effect. The residual effect impacts the testing for the interaction and causes the inflation (see Discussion for more details). For those four SNPs, we switched their main effect coding from an additive model to a 2 degree of freedom unrestricted coding and observed that the inflation factor for the interaction GxG meta-analysis results was diminished (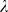
≤1.01).

## Supporting Information

Figure S1 **Simulation results comparing the performance of ARDI (red bars), multiplicative interaction model (black bars), and unrestricted interaction model (blue bars).** For each model, the barplots show the power (type I error for Model 1) of each method under different parameter settings.
(DOCX)

Figure S2 **ENCODE Integrated Regulation Tracks for 5q21.**
(DOCX)

Table S1 **An illustration of six two-SNP interaction models used in the simulation.** SNP 1 has genotype AA, Aa and aa; SNP 2 has genotype BB, Bb, and bb. A and B are the major alleles for SNP1 and 2, respectively. Each entry in the tables represents the risk of the corresponding genotype combination.
(DOCX)

Table S2 **Tools for functional annotation of non-coding variants.**
(DOCX)

Text S1 (DOCX)
