# Supplementary material for: Genome-Wide Search for Gene-Gene Interactions in Colorectal Cancer
Source: PLoS One. 2012 Dec 26;7(12):e52535. doi: 10.1371/journal.pone.0052535 (PMC3530500; doi:10.1371/journal.pone.0052535)
Supplement: Figure S1 — Simulation results comparing the performance of ARDI (red bars), multiplicative interaction model (black bars), and unrestricted interaction model (blue bars). For each model, the barplots show the power (type I error for Model 1) of each method under different parameter settings. (DOCX) [file pone.0052535.s001.docx]

Figure S1. Simulation results comparing the performance of ARDI (red bars), multiplicative interaction model (black bars), and unrestricted interaction model (blue bars). For each model, the barplots show the power (type I error for Model 1) of each method under different parameter settings.
