# Supplementary material for: Genome-Wide Search for Gene-Gene Interactions in Colorectal Cancer
Source: PLoS One. 2012 Dec 26;7(12):e52535. doi: 10.1371/journal.pone.0052535 (PMC3530500; doi:10.1371/journal.pone.0052535)
Supplement: Figure S2 — ENCODE Integrated Regulation Tracks for 5q21. (DOCX) [file pone.0052535.s002.docx]

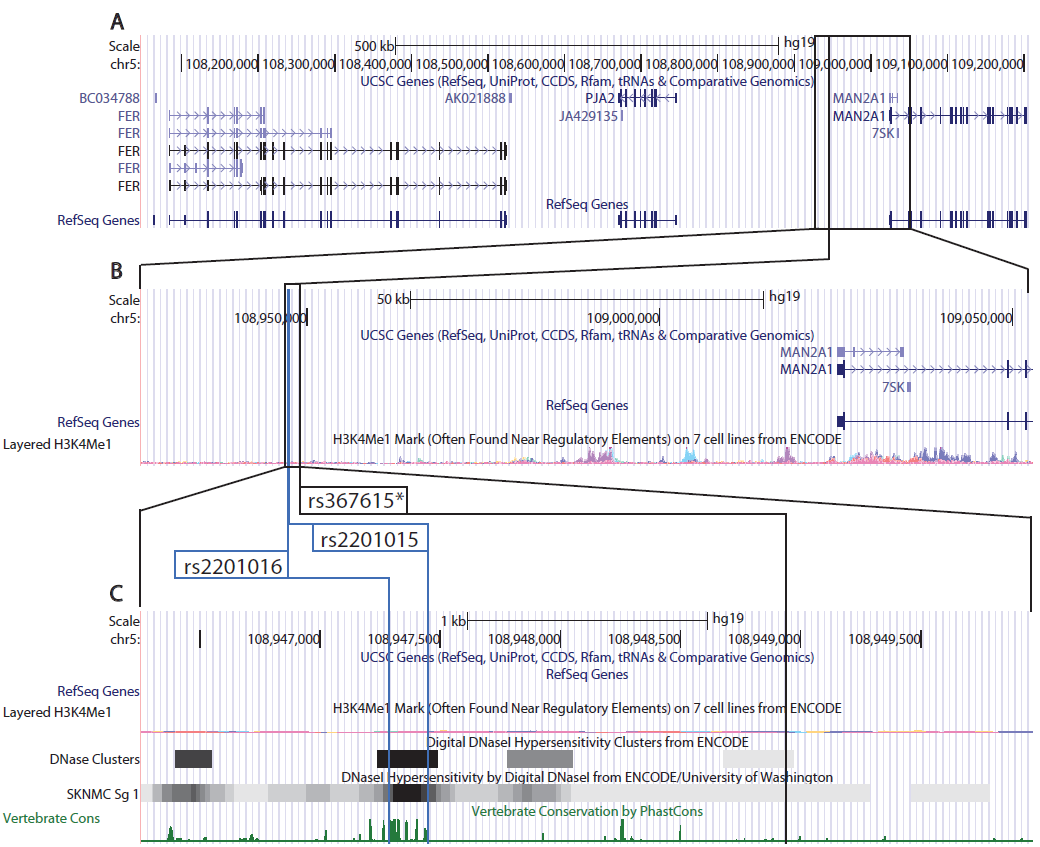


**Figure S2: ENCODE Integrated Regulation Tracks for 5q21. (A)** The UCSC Genome Browser view of chr5:108,047,215-109,211,548 (build 37) containing MAN2A1, PJA2, and FER. The vertical line represents the region containing susceptibility tagSNP rs367615 and two putative functional SNPs (rs2201016 and rs2201015) that are in high LD with rs367615 having r^2^ values of 1 and 0.916 respectively. The UCSC Gene track shows multiple variant transcripts for FER and MAN2A1. **(B)** The UCSC Genome Browser image of chr5:108,926,523-109,052,907 (build 37) and is a magnified view of the boxed region in (A). Shown directly beneath the Gene track in (B) is a transparent overlay of seven cell lines assayed by the ENCODE project displaying H3K4me1 marks. This track indicates relatively weak regulatory evidence in the large intergenic region harbouring rs36715. However, further magnification of this region, as shown in **(C)** (UCSC Genome Browser image of chr5:108,946,257-108,949,967), reveals that rs2201016 and rs2201015 fall within a region of strong DNase hypersensitivity and evolutionary conservation. Despite weak evidence from histone modification marks, the DNase Hypersensity clusters offer a more precise demarcation of chromatin accessibility. As shown in the track below ENCODE DNase clusters we see that this DNase hypersensitivity is found in the SKNMC cell line (neuroepithelioma derived from metastatic brain tumor). Lastly, shown below the DNase tracks is phastCons vertebrate 46-species alignment track showing rs2201016 and rs2201015 near a highly evolutionarily constrained region. Taken together, this evidence suggests that this region may be an enhancer for *FER*, *PJA2*, the closest gene *MAN2A1* or a more distal enhancer for *APC* (not shown).
