# Supplementary material for: Genome-Wide Search for Gene-Gene Interactions in Colorectal Cancer
Source: PLoS One. 2012 Dec 26;7(12):e52535. doi: 10.1371/journal.pone.0052535 (PMC3530500; doi:10.1371/journal.pone.0052535)
Supplement: Table S1 — An illustration of six two-SNP interaction models used in the simulation. SNP 1 has genotype AA, Aa and aa; SNP 2 has genotype BB, Bb, and bb. A and B are the major alleles for SNP1 and 2, respectively. Each entry in the tables represents the risk of the corresponding genotype combination. (DOCX) [file pone.0052535.s003.docx]

Table S1. An illustration of six two-SNP interaction models used in the simulation. SNP 1 has genotype AA, Aa and aa; SNP 2 has genotype BB, Bb, and bb. A and B are the major alleles for SNP1 and 2, respectively. Each entry in the tables represents the risk of the corresponding genotype combination.

| Model 1 | AA | Aa | aa |
| --- | --- | --- | --- |
| BB | $\alpha$ | $\alpha\times OR$ | $\alpha\times OR^{2}$ |
| Bb | $\alpha\times OR$ | $\alpha\times OR^{2}$ | $\alpha\times OR^{3}$ |
| bb | $\alpha\times OR^{2}$ | $\alpha\times OR^{3}$ | $\alpha\times OR^{4}$ |

| Model 2 | AA | Aa | aa |
| --- | --- | --- | --- |
| BB | $\alpha$ | $\alpha$ | $\alpha$ |
| Bb | $\alpha$ | $\alpha\times OR$ | $\alpha\times OR^{2}$ |
| bb | $\alpha$ | $\alpha\times OR^{2}$ | $\alpha\times OR^{4}$ |

| Model 3 | AA | Aa | aa |
| --- | --- | --- | --- |
| BB | $\alpha$ | $\alpha$ | $\alpha$ |
| Bb | $\alpha$ | $\alpha\times OR$ | $\alpha\times OR$ |
| bb | $\alpha$ | $\alpha\times OR$ | $\alpha\times OR$ |

| Model 4 | AA | Aa | aa |
| --- | --- | --- | --- |
| BB | $\alpha$ | $\alpha\times OR$ | $\alpha\times OR$ |
| Bb | $\alpha\times OR$ | $\alpha$ | $\alpha$ |
| bb | $\alpha\times OR$ | $\alpha$ | $\alpha$ |

| Model 5 | AA | Aa | aa |
| --- | --- | --- | --- |
| BB | $\alpha$ | $\alpha\times OR$ | $\alpha$ |
| Bb | $\alpha\times OR$ | $\alpha$ | $\alpha\times OR$ |
| bb | $\alpha$ | $\alpha\times OR$ | $\alpha$ |

| Model 6 | AA | Aa | aa |
| --- | --- | --- | --- |
| BB | $\alpha$ | $\alpha$ | $\alpha$ |
| Bb | $\alpha$ | $\alpha\times e^{uniform(0,\log(2))}$ | $\alpha\times e^{uniform(0,\log(2))}$ |
| bb | $\alpha$ | $\alpha\times e^{uniform(0,\log(2))}$ | $\alpha\times e^{uniform(0,\log(2))}$ |

| Model 3a | AA | Aa | aa |
| --- | --- | --- | --- |
| BB | $\alpha$ | $\alpha$ | $\alpha$ |
| Bb | $\alpha$ | $\alpha\times OR$ | $\alpha\times OR^{-1}$ |
| bb | $\alpha$ | $\alpha\times OR$ | $\alpha\times OR$ |

| Model 3b | AA | Aa | aa |
| --- | --- | --- | --- |
| BB | $\alpha$ | $\alpha$ | $\alpha$ |
| Bb | $\alpha$ | $\alpha\times OR$ | $\alpha\times OR^{-1}$ |
| bb | $\alpha$ | $\alpha\times OR$ | $\alpha\times OR^{-1}$ |
