# Supplementary material for: Genome-Wide Search for Gene-Gene Interactions in Colorectal Cancer
Source: PLoS One. 2012 Dec 26;7(12):e52535. doi: 10.1371/journal.pone.0052535 (PMC3530500; doi:10.1371/journal.pone.0052535)
Supplement: Table S2 — Tools for functional annotation of non-coding variants. (DOCX) [file pone.0052535.s004.docx]

**Table S2. Tools for functional annotation of non-coding variants**

| **UCSC Genome Browser** | **Genomic Class** | **Description** | **Functional evidence** |
| --- | --- | --- | --- |
| ENCODE Transcription | Transcribed region | Transcription levels in 7 Cell Lines from ENCODE. Assayed by high throughput sequencing of polyadenylated RNA. | Variable expression in different tissues provides evidence for cell type specific regulation when displayed as transparent overlay of each cell line. |
| ENCODE Layered H3K4Me1 | Non-Promoter regulatory elements | Uses ChIP-seq method to identify regions of DNA that interact with the mono-methylation of lysine 4 of the H3 histone protein in 7 different cell lines. Actual enhancer is likely a small portion of the broad region marked. | Methylation of histone proteins changes chromatin accessibility for transcription. H3K4Me1 is associated with enhancers downstream of transcription start site. |
| ENCODE Layered H3K4Me3 | Promoter regulatory element | Uses ChIP-seq method to identify regions of DNA that interact with the tri-methylation of lysine 4 of the H3 histone protein in 7 different cell lines. Actual regulatory element is likely a small portion of the broad region marked. | H3K4Me3 is associated with promoters that are active or accessible for activation. |
| ENCODE Layered H3K27Ac | Non-Promoter regulatory elements | Uses ChIP-seq method to identify regions of DNA that interact with the acetylation of lysine 27 of the H3 histone protein in 7 different cell lines. Actual regulatory element is likely a small portion of the broad region marked. | H3K27Ac enhances transcription possibly by blocking the spread of the repressive histone mark H3K27Me3. This mark is often Found Near Active Regulatory Elements |
| ENCODE DNase Clusters | Regulatory element | Measures digital DNaseI Hypersensitivity Clusters in a large collection of cell types from ENCODE. Greater precision than histone modifications. | Regulatory regions and promoters are susceptible DNase cutting. Hypersensitiviy is used to map chromatin accessibility. |
| ENCODE Txn Factor ChIP | Regulatory element | Transcription Factor ChIP-seq from ENCODE is assayed by Chromatin immunoprecipitation using antibodies for specific transcription factors and sequencing the precipitated DNA. | Marks regions where transcription factors bind DNA and exert specific functions. Activators can recruit RNA polymerase, repressors suppress recruitment, and insulators block the activity of nearby activators or repressors. |
| ENCODE UW CTCF Binding (Within the ENCODE Transcription Factor Binding Tracks) | Insulated Element | CTCF Binding Sites is assayed by chromatin immunoprecipitation using antibodies for CTCF and sequencing the precipitated DNA. | CTCF can function as a transcriptional activator, a repressor/silencer or an insulator. Binds chromatin insulators to prevent interaction between promoter and nearby enhancers or silencers. Also mediates long-range chromatin looping which can bring enhancers in proximity of a gene’s promoter. |
| Vertebrate Multi Alignment & Conservation (phastCons) | Conserved Element | Multiple alignments of 46 vertebrate species. Estimates the probability that each nucleotide belongs to a conserved element | Identification of evolutionarily conserved segments of homology, potentially identifying a functionally important region. |
