# Supplementary material for: Genome-Wide Search for Gene-Gene Interactions in Colorectal Cancer
Source: PLoS One. 2012 Dec 26;7(12):e52535. doi: 10.1371/journal.pone.0052535 (PMC3530500; doi:10.1371/journal.pone.0052535)
Supplement: Text S1 — (DOCX) [file pone.0052535.s005.docx]

**SIMULATION**

We considered six two-locus epistasis models (the first six models in Table S1) to compare the performance of ARDI with the multiplicative interaction model method and the unrestricted interaction model method illustrated in Table 2 of the main text. Models 1-3 are adopted from [1], where Model 1 is a null interaction model for evaluating the type I error, Model 2 is a two-locus interaction model with multiplicative effects and Model 3 is a two-locus interaction with threshold effects. Model 4 is an epistasis model adopted from [2,3] that has also been used to describe handedness in [4] and the color of swine in [5]. Model 5 is a classic exclusive OR model adopted from [3]. In Model 6, we tried to mimic the real application where interaction pattern is unknown. We generated the interaction effect size beta’s from a uniform distribution between 0 and log(2), representing interaction OR from 1 to 2. For each of Models 1-6, we generated the genotypes of two SNPs with MAF combinations = (0.2, 0.2), (0.4, 0.4), (0.05, 0.05), (0.1, 0.1), (0.2, 0.4) or (0.05, 0.1). A population of 1,000,000 was generated based on the corresponding model with rare disease prevalence $\alpha=\exp(-5)\approx0.0067$ and OR=1.1, 1.3 or 1.5 (except for Model 6 where OR is randomly generated between 1 and 2). Then 1000:1000 case-control samples were randomly selected from this population of 1000,000. For each parameter setting, we replicated the above procedure 1,000 times and calculate the power (Type I error rate for Model 1) under p-value threshold 0.05.

The results are summarized in the first three parts of Figure S1. The first part is for MAF combinations (0.2, 0.2) and (0.4, 0.4); the second part is for MAF combinations (0.05, 0.05) and (0.1, 0.1); the third part is for MAF combinations (0.05, 0.1) and (0.2, 0.4). In the first part, we can see from Model 1 that the type I error rates for all three methods are well controlled at the nominal level 0.05 (the horizontal line). In Model 2, multiplicative interaction method is the most powerful one because the underlying model is multiplicative. In Model 3 and 4, ARDI is the most powerful method as the interaction effect sizes are consistent for different genotype combinations. For Model 5, simple calculation shows that only the Aa and Bb combination has a non-zero interaction effect and in this case, the unrestricted interaction model is most powerful. In Model 6 where the true interaction pattern is assumed to be unknown, ARDI outperforms the other two methods. From the second part of Figure S1 where two SNPs are less common, we can see that the power differences between ARDI and multiplicative model get smaller. Unrestricted model performs considerably worse compared to the other two methods. It also has very conservative type I error rate when both SNPs have MAF 0.05, which is likely due to the small sample sizes in the combination of two homozygous minor genotypes. For the third part of Figure S1, previous conclusions from the first and second part generally still hold.

As we can see, the interaction effects for different genotype combinations are in the same direction in each of Models 1-6. If both negative and positive interaction effects exist, the performance of ARDI should be affected as it estimates the average interaction effect. Therefore, we modified Model 3 such that interaction effects can have different directions. The modified models are Model 3a and Model 3b (last two models in Table S1). Same simulation steps were then applied to the two modified models and the results were summarized in the fourth part of Figure S1. As expected, the advantage of ARDI compared with the unrestricted model in Model 3 is attenuated, sometimes even reversed, in the modified models Model 3a and Model 3b.

In summary, the performances of the three methods largely depend on the true underlying model and no method is uniformly better than others. Nonetheless, ARDI seems to have the advantage while the true underlying model is unknown.

**DESCRIPTION OF STUDY POPULATIONS INCUDED IN GWAS AND REPLICATION**

***French Association Study Evaluating RISK for sporadic colorectal cancer (ASTERISK)*** [6]. Participants were recruited from the Pays de la Loire region in France between December 2002 and March 2006. Eligibility criteria for cases included being of Caucasian origin, being greater than or 40 years of age at diagnosis, and having no family history of colorectal cancer or polyps. Cases were patients with first primary colorectal cancer diagnosed in one of the six public hospitals and five clinics located in the Pays de la Loire region which participated in the study. Cases were confirmed based on medical and pathology reports. Age- and sex-matched controls were recruited at two Health Examination Centers of the Pays de la Loire region, and the recruitment of controls greater than or 70 years was completed in the departments of internal medicine and hepatogastroenterology of the University Hospital Center of Nantes, located in the same region. Controls were eligible to participate if they were Caucasian, aged greater than or 40 years, and had no family history of colorectal cancer or polyps. In the presence of the physician, each participant filled out a standardized questionnaire on family information, medical history, lifestyle, and dietary intake. Cases and controls provided a blood sample.

***Colon Cancer Family Registry (CCFR)***. The CCFR is an NCI-supported consortium consisting of six centers dedicated to the establishment of a comprehensive collaborative infrastructure for interdisciplinary studies in the genetic epidemiology of colorectal cancer [7]. The CCFR includes data from approximately 30,500 total subjects (10,500 probands, and 20,000 unaffected and affected relatives and unrelated controls). Cases and controls, age 20 to 74 years, were recruited at the six participating centers beginning in 1998. CCFR implemented a standardized questionnaire that is administered to all participants, and includes established and suspected risk factors for colorectal cancer, which includes questions on medical history and medication use, reproductive history (for female participants), family history, physical activity, demographics, alcohol and tobacco use, and dietary factors. The Set 1 scan, which has been described previously [8] (Figueiredo JC et al 2011), includes population-based cases and age-matched controls from the three population-based centers: Seattle, Toronto and Australia. Cases were genetically enriched by over-sampling those with a young age at onset or positive family history. Controls were matched to cases on age and sex. All cases and controls were self-reported as White, which was confirmed with genotype data. The Set 2 scan includes population-based cases and matched controls from all six Colon CFR centers including Mayo Clinic, Hawaii, University of Southern California, Seattle, Toronto and Australia. As with Set 1, cases were genetically enriched by over-sampling those with a young age at onset or positive family history. Controls were same generation family controls.

***Hawai’i Colorectal Cancer Studies 2 & 3 (Colo2&3)****[9] (Le Marchand 2001).* Patients with colorectal cancer were identified through the rapid reporting system of the Hawaii SEER registry and consisted of all Japanese, Caucasian, and Native Hawaiian residents of Oahu who were newly diagnosed with an adenocarcinoma of the colon or rectum between January 1994 and August 1998. Control subjects were selected from participants in an on-going population-based health survey conducted by the Hawaii State Department of Health and from Health Care Financing Administration participants. Controls were matched to cases by sex, ethnicity, and age (within two years). Personal interviews were obtained from 768 matched pairs, resulting in a participation rate of 58.2% for cases and 53.2% for controls. A questionnaire, administered during an in-person interview, included questions about demographics, lifetime history of tobacco, alcohol use, aspirin use, physical activity, personal medical history, family history of colorectal cancer, height and weight, diet (FFQ), and postmenopausal hormone use. A blood sample was obtained from 548 (71%) of interviewed cases and 662 (86%) of interviewed controls. SEER staging information was extracted from the Hawaii Tumor Registry. In GECCO, self-reported Caucasian subjects with DNA, and clinical and epidemiologic data were selected for genotyping.

***Darmkrebs: Chancen der Verhütung durch Screening (DACHS)*** [10,11]. This German study was initiated as a large population-based case-control study in 2003 in the Rhine-Neckar-Odenwald region (southwest region of Germany) to assess the potential of endoscopic screening for reduction of colorectal cancer risk and to investigate etiologic determinants of disease, particularly lifestyle/environmental factors and genetic factors. Cases with a first diagnosis of invasive colorectal cancer (ICO-10 codes C18-C20) who were at least 30 years of age (no upper age limit), German speaking, a resident in the study region, and mentally and physically able to participate in a one-hour interview, were recruited by their treating physicians either in the hospital a few days after surgery, or by mail after discharge from the hospital. Cases were confirmed based on histologic reports and hospital discharge letters following diagnosis of colorectal cancer. All hospitals treating colorectal cancer patients in the study region participated. Based on estimates from population-based cancer registries, more than 50% of all potentially eligible patients with incident colorectal cancer in the study region were included. The distribution of key socio-demographic and clinical factors is similar to that reported form population based cancer registries. Community-based controls were randomly selected from population registries, employing frequency matching with respect to age (5-year groups), sex, and county of residence. Controls with a family history of colorectal cancer were excluded. Controls were contacted by mail and follow-up calls. The participation rate was 51%. During an in-person interview, data were collected on demographics, medical history, family history of CRC, and various life-style factors, as were blood, saliva, and mouthwash samples. The Set 1 scan consisted of a subset of participants recruited by the time of first genotyping in 2009, and samples were frequency matched on age and gender. The Set 2 scan consisted of additional subjects that were later recruited as part of this ongoing study.

***Diet, Activity, and Lifestyle Study (DALS)****[12]****.*** DALS is a population-based case-control study of colon cancer. Participants were recruited between 1991 and 1994 from three locations: the Kaiser Permanente Medical Care Program (KPMCP) of Northern California, an eight-county area in Utah, and the metropolitan Twin Cities area of Minnesota. Eligibility criteria for cases included age at diagnosis between 30 and 79 years, diagnosis with first primary colon cancer (ICD-O-2 codes 18.0 and 18.2-18.9) between October 1^st^ 1991 and September 30^th^ 1994, English speaking, and competency to complete the interview. Individuals with cancer of the rectosigmoid junction or rectum were excluded, as were those with a pathology report noting familial adenomatous polyposis, Crohn’s disease, or ulcerative colitis. A rapid-reporting system was used to identify all incident cases of colon cancer resulting in the majority of cases being interviewed within four months of diagnosis. Controls from KPMCP were randomly selected from membership lists. In Utah, controls under 65 years of age were randomly selected through random-digit dialing and driver license lists. Controls, 65 years of age and older, were randomly selected from Health Care Financing Administration lists. In Minnesota, controls were identified from Minnesota driver’s license or state ID lists. Cases and controls were matched to cases by 5-year age groups and sex. The Set I scan consisted of a subset of the study designed above, from Utah, Minnesota, and KPMCP, and was restricted to subjects who self-reported as White non-Hispanic. The Set 2 scan consisted of subjects from Utah and Minnesota that were not genotyped in Set 1. Set 2 was restricted to subjects who self-reported as White non-Hispanic and those that had appropriate consent to post data to dbGaP.

***Health Professionals Follow-up Study (HPFS)***(Rimm et al., 1990). The HPFS is a parallel prospective study to the Nurses’ Health Study (NHS). The HPFS cohort comprises 51,529 men who, in 1986, responded to a mailed questionnaire. The participants are U.S. male dentists, optometrists, osteopaths, podiatrists, pharmacists, and veterinarians born between 1910 and 1946. Participants have provided information on health related exposures, including: current and past smoking history, age, weight, height, diet, physical activity, aspirin use, and family history of colorectal cancer. Colorectal cancer and other outcomes were reported by participants or next-of-kin and followed up through review of the medical and pathology record by physicians. Overall, more than 97% of self-reported colorectal cancers were confirmed by medical record review. Information was abstracted on histology and primary location. Incident cases are defined as those occurring after the subject provided the blood sample. Prevalent cases are defined as those occurring after enrollment in the study, but prior to the subject providing the blood sample. Follow-up has been excellent, with 94% of the men responding to date. Colorectal cancer cases were ascertained through January 1, 2008. After excluding participants with histories of cancer (except non-melanoma skin), ulcerative colitis, or familial polyposis, two case-control sets were constructed: 1) a case-control set with cases of colorectal cancer matched to randomly selected controls who provided a blood sample and were free of colorectal cancer at the same time the colorectal cancer was diagnosed in the cases; 2) a case-control set with cases of colorectal cancer matched to randomly selected controls who provided a buccal sample and were free of colorectal cancer at the same time the colorectal cancer was diagnosed in the case. For both case-control sets, matching criteria included year of birth (within 1 year) and month/year of blood or buccal cell sampling (within six months). Cases were pair matched 1:1, 1:2, or 1:3 with a control participant(s).

In addition to colorectal cancer cases and controls, a set of adenoma cases and matched controls were selected for genotyping. Over follow-up, data were collected on endoscopic screening practices and, if individuals have been diagnosed with polyp, the polyps were confirmed to be adenomatous by medical record review. Adenoma cases were ascertained through January 1, 2008. A separate case-control set was constructed of participants diagnosed with advanced adenoma matched to control participants who underwent a lower endoscopy in the same time period and did not have an adenoma. Advanced adenoma was defined as an adenoma > 1 cm in diameter and / or with tubulovillous, villous, or high-grade dysplasia / carcinoma-in-situ histology. Matching criteria included year of birth (within one year) and month/year of blood sampling (within six months), the reason for their lower endoscopy (screening, family history, or symptoms) and the time period of any prior endoscopy (within two years).

***Multiethnic Cohort Study (MEC)(20)*** (Kolonel 2000). MEC was initiated in 1993 to investigate the impact of dietary and environmental factors on major chronic diseases, particularly cancer, in ethnically diverse populations in Hawai’i and California. The study recruited 96,810 men and 118,441 women aged 45 to 75 years between 1993 and 1996. Incident colorectal cancer cases occurring since January 1995, and controls were contacted for blood or saliva samples. The median interval between diagnosis and blood draw was 14 months (interquartile range, 10-19) among cases and the participation rate 74%. A sample of cohort participants was randomly selected to serve as controls at the onset of the nested case-control study (participation rate 66%). The selection was stratified by sex, age, and race/ethnicity. Colorectal cancer cases are identified through the Rapid Reporting System of the Hawai’i Tumor Registry and through quarterly linkage to the Los Angeles County Cancer Surveillance Program. Both registries are members of SEER. In GECCO, self-reported White subjects from the nested case-control study described above with DNA, and clinical and epidemiologic data were selected for genotyping

***Nurses’ Health Study (NHS)***(Belanger, Hennekens, Rosner, & Speizer, 1978). The NHS cohort began in 1976 when 121,700 married female registered nurses aged 30 to 55 years returned the initial questionnaire that ascertained a variety of important health-related exposures. Since 1976, follow-up questionnaires have been mailed every two years. Colorectal cancer and other outcomes were reported by participants or next-of-kin and followed up through review of the medical and pathology record by physicians. Incident cases are defined as those occurring after the subject provided the blood sample. Prevalent cases are defined as those occurring after enrollment in the study in 1976, but prior to the subject providing the blood sample. Overall, more than 97% of self-reported colorectal cancers were confirmed by medical-record review. Information was abstracted on histology and primary location. Follow-up has been high: as a proportion of the total possible follow-up time, follow-up has been over 92%. Colorectal cancer cases were ascertained through June 1, 2008. After excluding participants with histories of cancer (except non-melanoma skin), ulcerative colitis, or familial polyposis, we constructed two case-control sets: 1) a case-control set with cases of colorectal cancer matched to randomly selected controls who provided a blood sample and were free of colorectal cancer at the same time the colorectal cancer was diagnosed in the case; 2) a case-control set with cases of colorectal cancer matched to randomly selected controls who provided a buccal sample and were free of colorectal cancer at the same time the colorectal cancer was diagnosed in the cases. For both case-control sets, matching criteria included year of birth (within one year) and month / year of blood or buccal cell sampling (within six months). Cases were pair matched 1:1, 1:2, or 1:3 with a control participant(s).

In addition to colorectal cancer cases and controls, a set of adenoma cases and matched controls were selected for genotyping. Over follow-up, data were collected on endoscopic screening practices and, if individuals have been diagnosed with polyp, the polyps confirmed to be adenomatous by medical record review. Adenoma cases were ascertained through June 1, 2008. A separate case-control set was constructed of participants diagnosed with advanced adenoma matched to control participants who underwent a lower endoscopy in the same time period and did not have an adenoma. Advanced adenoma was defined as an adenoma > 1 cm in diameter and / or with tubulovillous, villous, or high-grade dysplasia / carcinoma-in-situ histology. Matching criteria included year of birth (within one year) and month/year of blood sampling (within six months), the reason for their lower endoscopy (screening, family history, or symptoms) and the time period of any prior endoscopy (within two years).

***Ontario Registry for Studies of Familial Colorectal Cancer (OFCCR).*** In GECCO, a subset of the Assessment of Risk in Colorectal Tumours in Canada (ARCTIC) from the Ontario Registry for Studies of Familial Colorectal Cancer (OFCCR) was used. Both the case-control study [13] and the OFCCR[14] have been described in detail previously, as have GWAS results[15]. In brief, cases were confirmed incident colorectal cancer (CRC) cases ages 20 to 74 years, residents of Ontario identified through comprehensive registry and diagnosed between July 1997 and June 2000. Population-based controls were randomly selected among Ontario residents (random-digit-dialing and listing of all Ontario residents), and matched by sex and 5-year age groups. A total of 1,236 CRC cases and 1,223 controls were successfully genotyped on at least one of the Illumina 1536 GoldenGate assay, the Affymetrix GeneChip® Human Mapping 100K and 500K Array Set, and a 10K non-synonymous SNP chip. Analysis was based on a set of unrelated subjects who were found to be non-Hispanic, White by self-report or by investigation of genetic ancestry. We further excluded subjects if there was a sample mix-up, if they were missing epidemiologic questionnaire data, if they were appendix cases, or if they overlapped with the Colon Cancer Family Registry. Additionally, only samples genotyped on the Affymetrix GeneChip® 500K Array were utilized in order to avoid coverage issues in imputation.

***Physician’s Health Study (PHS****)*(Hennekens & Eberlein, 1985; Christen, Gaziano, & Hennekens, 2000). The PHS was established as a randomized, double-blind, placebo-controlled trial of aspirin and ß-carotene among 22,071 healthy U.S. male physicians, between 40 and 84 years of age in 1982. Participants completed two mailed questionnaires before being randomly assigned, additional questionnaires at six and 12 months, and questionnaires annually thereafter. In addition, participants were sent postcards at six months to ascertain status. From August 1982 to December 1984, 14,916 baseline blood samples were collected from the physicians during the run-in phase before randomization. When participants report a diagnosis of cancer, medical records and pathology reports are reviewed by study physicians who are blinded to exposure data. Among those who provided baseline blood samples, colorectal cases were ascertained through March 31, 2008, and controls were matched on age (within one year for younger participants, up to five years for older participants) and smoking status (never, past, current). Cases were “pair” matched 1:1, 1:2 or 1:3 with a control participant(s). Two sets of samples were genotyped on the same platform at the same genotyping center at separate points in time.

***Prostate, Lung, Colorectal, and Ovarian Cancer Screening Trial (PLCO)***. PLCO enrolled 154,934 participants (men and women, aged between 55 and 74 years) at ten centers into a large, randomized, two-arm trial to determine the effectiveness of screening to reduce cancer mortality. Sequential blood samples were collected from participants assigned to the screening arm. Participation was 93% at the baseline blood draw. In the observational (control) arm, buccal cells were collected via mail using the “swish-and-spit” protocol and participation rate was 65%. Details of this study have been previously described[16,17] (Prorok 2000, Gohagan 2000) and are available online (http://dcp.cancer.gov/plco).

The Set 1 scan included a subset of 577 colon cancer cases self-reported as being non-Hispanic White with available DNA samples, questionnaire data, and appropriate consent for ancillary epidemiologic studies. Cases were excluded if they had a history of inflammatory bowel disease, polyps, polyposis syndrome or cancer (excluding basal or squamous cell skin cancer). Controls come from the Cancer Genetic Markers of Susceptibility (CGEMS) prostate cancer scan [18,19] (all male) and the GWAS of Lung Cancer and Smoking[20] (enriched for smokers) along with an additional 92 non-Hispanic White female controls. For the Set 2 scan, cases were colorectal cancers from both arms of the trail, which were not already included in Set 1. Samples were excluded if participants did not sign appropriate consents, if DNA was unavailable, if baseline questionnaire data with follow-up were unavailable, if they had a history of colon cancer prior to the trial, if they were a rare cancer, and if they were already in colon GWAS, or if they were a control in the prostate or lung populations. Controls were frequency matched 1:1 to cases without replacement, and cases were not eligible to be controls. Matching criteria were age at enrollment (two year blocks), enrollment date (two year blocks), sex, race / ethnicity, trial arm, and study year of diagnosis (i.e. controls must be cancer free into the case's year of diagnosis).

***Postmenopausal Hormones Supplementary Study to the Colon Cancer Family Registry (PMH-CCFR*)**[21]. Eligible case patients included all female residents, ages 50 to 74 years, residing in the 13 counties in Washington State reporting to the Cancer Surveillance SEER program, who were newly diagnosed with invasive colorectal adenocarcinoma (ICD-O C18.0, C18.2-.9, C19.9, C20.0-.9) between October 1998 and February 2002. Eligibility for all individuals was limited to those who were English-speaking with available telephone numbers, in which they could be contacted. On average, cases were identified within four months of diagnosis. The overall response proportion of eligible cases identified was 73%. Community-based controls were randomly selected according to age distribution (in 5-year age intervals) of the eligible cases by using lists of licensed drivers from the Washington State Department of Licensing for individuals, ages 50 to 64 years, and rosters from the Health Care Financing Administration (now the Centers for Medicare and Medicaid) for individuals older than 64 years. The overall response proportion of eligible controls was 66%. In GECCO, samples with sufficient DNA and with DNA extracted from blood were genotyped. Only participants that were not part of the CCFR Seattle site were included in the sample set.

***VITamins And Lifestyle (VITAL).*** The VITamins And Lifestyle (VITAL) cohort comprises 77,721 Washington State men and women aged 50 to 76 years, recruited from 2000 to 2002 to investigate the associations of supplement use and lifestyle factors with cancer risk. Subjects were recruited by mail, from October 2000 to December 2002, using names purchased from a commercial mailing list. All subjects competed a 24 page questionnaire and buccal-cell specimens for DNA was self-collected by 70% of the participants. Subjects are followed for cancer by linkage to the western Washington SEER cancer registry and are censored when they move out of the area covered by the registry or at time of death. Details of this study have been previously described [22]. In GECCO, a nested case-control set was genotyped. Samples included, colorectal cancer cases with DNA, excluding subject with colorectal cancer before baseline, in situ cases, (large cell) neuroendocrine carcinoma, squamous cell carcinoma, carcinoid tumor, Goblet cell carcinoid, any type of lymphoma, including non-Hodgkin, Mantle cell, large B-cell, or follicular lymphoma. Controls were matched on age at enrollment (within one year), enrollment date (within one year), sex, and race / ethnicity. One control was randomly selected per case among all controls that matched on the four factors above and where the control follow-up time was greater than follow-up time of the case.

**Women’s Health Initiative (WHI).** WHI is a long-term health study of 161,808 post-menopausal women aged 50 to 79 years at 40 clinical centers throughout the U.S. WHI comprises a Clinical Trial (CT) arm, an Observational Study (OS) arm, and several extension studies. The details of WHI have been previously described(31,32) (Hays 2003, WHI study group 1998) and are available online (https://cleo.whi.org/SitePages/Home.aspx). In GECCO, Set 1 cases were selected from the September 12, 2005 database and were comprised of centrally adjudicated colon cancer cases from the Observational Study (OS) who self-reported as White. Controls were first selected among controls previously genotyped as part of a Hip Fracture GWAS conducted within the WHI OS and matched to cases on age (within three years) enrollment date (within 365 days), hysterectomy status, and prevalent conditions at baseline. For 37 cases, there was not a control match in the Hip Fracture GWAS. For these participants, we identified a matched control in the WHI OS based on same criteria. In the Set 2 scan, cases were selected from the August 2009 database and were comprised of centrally adjudicated colon and colorectal cancer cases from the OS and CT who were not genotyped in Set 1. In addition, case and control participants were subject to the following exclusion criteria: a prior history of colorectal cancer at baseline, IRB approval not available for data submission into dbGaP, and not sufficient DNA available. Matching criteria included age (within years), race/ethnicity, WHI date (within three years), WHI Calcium and Vitamin D study date (within three years), and randomization arms (OS flag, hormone therapy assignments, dietary modification assignments, calcium/vitamin D assignments). In addition, Whites were matched on randomization center (40 centers). Each case was matched with one control (1:1) that exactly met the matching criteria. Control selection was done in a time-forward manner, selecting one control for each case first from the risk set at the time of the case’s event. The matching algorithm was allowed to select the closest match based on a criteria to minimize an overall distance measure(33) (Bergstralh EJ, Kosanke JL. Computerized matching of cases to controls. Technical Report #56, Department of Health Sciences Research, Mayo Clinic, Rochester MN. April 1995). Each matching factor was given the same weight.

**Description of Functional Annotations**

There are several bioinformatic tools available for the post-GWAS functional characterization of putative disease causing loci through the UCSC genome browser [23]. Annotation of non-protein-coding regions operates under the hypothesis that trait-associated alleles exert their effects by influencing transcriptional levels through multiple regulatory mechanisms. The UCSC genome browser provides several tracks that can be used to annotate enhancers, promoters, insulators and silencers (for details see Table S2). Such tools help expedite discovery of causal variants by isolating a few likely culprits from a large background of variants in linkage disequilibrium with the surrogate marker (tag SNP). Since distal enhancers often facilitate cell-type specific expression, it is helpful to look for evidence in a variety of cell lines in addition to those related to the trait. For example, the ENCODE Transcription summary track assayed by RNA-seq can be displayed as an overlay of histograms denoting expression levels in various tissues marked by a specific color, thus allowing identification of cell-type specific expression.

Similarly the histone modification tracks can provide additional evidence for cell-specific regulatory elements when displayed in this configuration. The methylation and acetylation of histone proteins changes chromatin accessibility for transcription and such marks can serve as a powerful tool for identifying both enhancer and promoter regions. There are three summary ENCODE tracks available to detect specific chemical modifications and were assayed in seven different tissues using ChIP-seq methodology. The H3K4me1 histone mark is associated with enhancers downstream of transcription start sites. The H3k27Ac histone mark is similarly thought to enhance transcription and likely does so through the blocking of the repressive histone mark H3K27Me3. The last histone modification in the summary tracks, H3K4Me3, is associated with active promoters. Additional chemical modifications and cell lines are available under the Broad Institute histone modification track for further interrogation.

Regulatory regions are susceptible to DNase cutting and ENCODE has assayed this hypersensitivity in a large collection of cell types. The precision of the DNase cluster track is somewhat better than that of chromatin modifications. Identification of evolutionarily conserved segments, ‘phylogenetic footprints’, has been used to discover functionally important regions. However, Histone marks and DNase hypersensitivity tracks are more robust tools for characterizing regulatory regions because these elements are not always constrained across vertebrate evolution. Functional hypotheses around regulatory regions can be strengthened with the ENCODE Transcription Factor track. Using the ChIP-seq method, this track helps identify the alteration of transcription factor binding sites, which potentially alter expression levels. As an example, CTCF is a transcription factor that assumes multiple forms and can act as an activator, a repressor/silencer or an insulator. When binding chromatic insulators it can prevent interactions between promoters and nearby enhancers or silencers. However it also mediates long-range chromatin looping which can bring enhancers in proximity of a gene’s promoter. Combining the strengths and weaknesses of each of these tracks can provide *in silico* evidence for regulatory function, and enables selection of strong candidates for additional functional studies using reporter gene methods.

Reference List

1. Marchini J, Donnelly P, Cardon LR (2005) Genome-wide strategies for detecting multiple loci that influence complex diseases. Nat Genet 37: 413-417.

2. Neuman RJ, Rice JP (1992) Two-locus models of disease. Genet Epidemiol 9: 347-365. 10.1002/gepi.1370090506 [doi].

3. Wan X, Yang C, Yang Q, Xue H, Fan X, Tang NL, Yu W (2010) BOOST: A fast approach to detecting gene-gene interactions in genome-wide case-control studies. Am J Hum Genet 87: 325-340. S0002-9297(10)00378-2 [pii];10.1016/j.ajhg.2010.07.021 [doi].

4. Levy J, Nagylaki T (1972) A model for the genetics of handedness. Genetics 72: 117-128.

5. Lerner, I. Michael (1968) Heredity, evolution, and society. San Francisco: W. H. Freeman.

6. Küry S, Buecher B, Robiou-du-Pont S, Scoul C, Sebille V, Colman H, Le HC, Le NT, Bourdon J, Faroux R, Ollivry J, Lafraise B, Chupin LD, Bezieau S (2007) Combinations of cytochrome P450 gene polymorphisms enhancing the risk for sporadic colorectal cancer related to red meat consumption. Cancer Epidemiol Biomarkers Prev 16: 1460-1467. 16/7/1460 [pii];10.1158/1055-9965.EPI-07-0236 [doi].

7. Newcomb PA, Baron J, Cotterchio M, Gallinger S, Grove J, Haile R, Hall D, Hopper JL, Jass J, Le ML, Limburg P, Lindor N, Potter JD, Templeton AS, Thibodeau S, Seminara D (2007) Colon Cancer Family Registry: an international resource for studies of the genetic epidemiology of colon cancer. Cancer Epidemiol Biomarkers Prev 16: 2331-2343.

8. Figueiredo JC, Lewinger JP, Song C, Campbell PT, Conti DV, Edlund CK, Duggan DJ, Rangrej J, Lemire M, Hudson T, Zanke B, Cotterchio M, Gallinger S, Jenkins M, Hopper J, Haile R, Newcomb P, Potter J, Baron JA, Marchand LL, Casey G (2011) Genotype-environment interactions in microsatellite stable/microsatellite instability-low colorectal cancer: results from a genome-wide association study. Cancer Epidemiol Biomarkers Prev 20: 758-766. 1055-9965.EPI-10-0675 [pii];10.1158/1055-9965.EPI-10-0675 [doi].

9. Le Marchand L, Hankin JH, Wilkens LR, Pierce LM, Franke A, Kolonel LN, Seifried A, Custer LJ, Chang W, Lum-Jones A, Donlon T (2001) Combined effects of well-done red meat, smoking, and rapid N-acetyltransferase 2 and CYP1A2 phenotypes in increasing colorectal cancer risk. Cancer Epidemiol Biomarkers Prev 10: 1259-1266.

10. Brenner H, Chang-Claude J, Seiler CM, Rickert A, Hoffmeister M (2011) Protection from colorectal cancer after colonoscopy: population-based case-control study. Ann Intern Med 154: 22-30.

11. Vossen CY, Hoffmeister M, Chang-Claude JC, Rosendaal FR, Brenner H (2011) Clotting factor gene polymorphisms and colorectal cancer risk. J Clin Oncol 29: 1722-1727. JCO.2010.31.8873 [pii];10.1200/JCO.2010.31.8873 [doi].

12. Slattery ML, Friedman GD, Potter JD, Edwards S, Caan BJ, Samowitz W (1996) A description of age, sex, and site distributions of colon carcinoma in three geographic areas. Cancer 78: 1666-1670.

13. Cotterchio M, Manno M, Klar N, McLaughlin J, Gallinger S (2005) Colorectal screening is associated with reduced colorectal cancer risk: a case-control study within the population-based Ontario Familial Colorectal Cancer Registry. Cancer Causes Control 16: 865-875.

14. Cotterchio M, Keown-Eyssen G, Sutherland H, Buchan G, Aronson M, Easson AM, Macey J, Holowaty E, Gallinger S (2000) Ontario familial colon cancer registry: methods and first-year response rates. Chronic Dis Can 21: 81-86.

15. Zanke BW, Greenwood CM, Rangrej J, Kustra R, Tenesa A, Farrington SM, Prendergast J, Olschwang S, Chiang T, Crowdy E, Ferretti V, Laflamme P, Sundararajan S, Roumy S, Olivier JF, Robidoux F, Sladek R, Montpetit A, Campbell P, Bezieau S, O'shea AM, Zogopoulos G, Cotterchio M, Newcomb P, McLaughlin J, Younghusband B, Green R, Green J, Porteous ME, Campbell H, Blanche H, Sahbatou M, Tubacher E, Bonaiti-Pellie C, Buecher B, Riboli E, Kury S, Chanock SJ, Potter J, Thomas G, Gallinger S, Hudson TJ, Dunlop MG (2007) Genome-wide association scan identifies a colorectal cancer susceptibility locus on chromosome 8q24. Nat Genet 39: 989-994.

16. Prorok PC, Andriole GL, Bresalier RS, Buys SS, Chia D, Crawford ED, Fogel R, Gelmann EP, Gilbert F, Hasson MA, Hayes RB, Johnson CC, Mandel JS, Oberman A, O'Brien B, Oken MM, Rafla S, Reding D, Rutt W, Weissfeld JL, Yokochi L, Gohagan JK (2000) Design of the Prostate, Lung, Colorectal and Ovarian (PLCO) Cancer Screening Trial. Control Clin Trials 21: 273S-309S.

17. Gohagan JK, Prorok PC, Hayes RB, Kramer BS (2000) The Prostate, Lung, Colorectal and Ovarian (PLCO) Cancer Screening Trial of the National Cancer Institute: history, organization, and status. Control Clin Trials 21: 251S-272S.

18. National Cancer Institute (2009) Cancer Genetic Markers of Susceptibility (CGEMS) data website.

19. Yeager M, Chatterjee N, Ciampa J, Jacobs KB, Gonzalez-Bosquet J, Hayes RB, Kraft P, Wacholder S, Orr N, Berndt S, Yu K, Hutchinson A, Wang Z, Amundadottir L, Feigelson HS, Thun MJ, Diver WR, Albanes D, Virtamo J, Weinstein S, Schumacher FR, Cancel-Tassin G, Cussenot O, Valeri A, Andriole GL, Crawford ED, Haiman CA, Henderson B, Kolonel L, Le ML, Siddiq A, Riboli E, Key TJ, Kaaks R, Isaacs W, Isaacs S, Wiley KE, Gronberg H, Wiklund F, Stattin P, Xu J, Zheng SL, Sun J, Vatten LJ, Hveem K, Kumle M, Tucker M, Gerhard DS, Hoover RN, Fraumeni JF, Jr., Hunter DJ, Thomas G, Chanock SJ (2009) Identification of a new prostate cancer susceptibility locus on chromosome 8q24. Nat Genet .

20. Landi MT, Chatterjee N, Yu K, Goldin LR, Goldstein AM, Rotunno M, Mirabello L, Jacobs K, Wheeler W, Yeager M, Bergen AW, Li Q, Consonni D, Pesatori AC, Wacholder S, Thun M, Diver R, Oken M, Virtamo J, Albanes D, Wang Z, Burdette L, Doheny KF, Pugh EW, Laurie C, Brennan P, Hung R, Gaborieau V, McKay JD, Lathrop M, McLaughlin J, Wang Y, Tsao MS, Spitz MR, Wang Y, Krokan H, Vatten L, Skorpen F, Arnesen E, Benhamou S, Bouchard C, Metsapalu A, Vooder T, Nelis M, Valk K, Field JK, Chen C, Goodman G, Sulem P, Thorleifsson G, Rafnar T, Eisen T, Sauter W, Rosenberger A, Bickeboller H, Risch A, Chang-Claude J, Wichmann HE, Stefansson K, Houlston R, Amos CI, Fraumeni JF, Jr., Savage SA, Bertazzi PA, Tucker MA, Chanock S, Caporaso NE (2009) A genome-wide association study of lung cancer identifies a region of chromosome 5p15 associated with risk for adenocarcinoma. Am J Hum Genet 85: 679-691. S0002-9297(09)00408-X [pii];10.1016/j.ajhg.2009.09.012 [doi].

21. Newcomb PA, Zheng Y, Chia VM, Morimoto LM, Doria-Rose VP, Templeton A, Thibodeau SN, Potter JD (2007) Estrogen plus progestin use, microsatellite instability, and the risk of colorectal cancer in women. Cancer Res 67: 7534-7539. 67/15/7534 [pii];10.1158/0008-5472.CAN-06-4275 [doi].

22. White E, Patterson RE, Kristal AR, Thornquist M, King I, Shattuck AL, Evans I, Satia-Abouta J, Littman AJ, Potter JD (2004) VITamins And Lifestyle cohort study: study design and characteristics of supplement users. Am J Epidemiol 159: 83-93.

23. Kent WJ, Sugnet CW, Furey TS, Roskin KM, Pringle TH, Zahler AM, Haussler D (2002) The human genome browser at UCSC. Genome Res 12: 996-1006. 10.1101/gr.229102. Article published online before print in May 2002 [doi].
